# A universal 2D-on-SiC platform for heterogeneous integration of epitaxial III-N membranes

**DOI:** 10.1126/sciadv.adz3605

**Published:** 2025-11-21

**Authors:** Se H. Kim, Hanjoo Lee, Dong-Gwan Kim, Donghan Kim, Seokgi Kim, Hyunsoo Kim, Seoyong Ha, Hyunho Yang, Yunsu Jang, Jangho Yoon, ByoungTak Lee, Jung-Hee Lee, Roy Byung Kyu Chung, Hongsik Park, Sungkyu Kim, Tae Hoon Lee, Hyun S. Kum

**Affiliations:** ^1^ Department of Electrical and Electronic Engineering, Yonsei University, Seoul, South Korea.; ^2^ Department of Advanced Materials Science and Engineering, Kyungpook National University, Daegu, South Korea.; ^3^ Department of Electronic and Electrical Engineering, Kyungpook National University, Daegu, South Korea.; ^4^ Department of Nanotechnology and Advanced Materials Engineering, Sejong University, Seoul, South Korea.; ^5^ LX Semicon Co. Ltd., 222, Techno 2-ro, Yuseong-gu, Daejeon, South Korea.; ^6^ L&D Co. Ltd., 179, Daehak-ro, Yuseong-gu, Daejeon, South Korea.

## Abstract

Nonconventional epitaxial techniques, such as van der Waals epitaxy and remote epitaxy, have attracted substantial attention in the semiconductor research community for their capability to repeatedly produce high-quality freestanding films from a single mother wafer. Successful implementation of these techniques depends on creating a robust, uniform two-dimensional (2D) material surface. The conventional method for fabricating graphene on silicon carbide (SiC) is high-temperature graphitization. However, the extremely high temperature required for silicon sublimation (typically above 1500°C) causes step bunching, forming nonuniform multilayer graphene stripes and an unfavorable surface morphology for epitaxial growth. Here, we developed a wafer-scale graphitization technique that allows fast synthesis of single-crystalline graphene at low temperatures by metal-assisted graphitization. In contrast to previous reports, we found annealing conditions enabling SiC dissociation while avoiding silicide formation, producing uniform single-crystalline graphene while maintaining the pristine surface morphology of the substrate. We successfully produce high-quality freestanding single-crystalline III-N (AlN and GaN) membranes on graphene/SiC via the 2D material–based layer transfer technique.

## Teaser

A universal 2D-on-SiC platform produces wafer-scale graphene and high-quality freestanding III-N membranes.

## INTRODUCTION

Two-dimensional (2D) material-based epitaxy techniques, such as van der Waals epitaxy (vdWE) and remote epitaxy, have garnered substantial attention due to their ability to address fundamental challenges inherent in conventional heteroepitaxial techniques caused by lattice and thermal expansion mismatches (*1*–*5*). The vdWE and remote epitaxial techniques not only alleviate these issues but also potentially allow reuse of costly semiconductor substrates for substantial cost reduction of electrical devices. These techniques enable precise exfoliation of highly functional single-crystalline membranes and allow heterogeneous integration of dissimilar materials, thereby enabling the integration of distinct electronic and photonic elements onto a single platform (*1*). Numerous studies on these epitaxial techniques have been investigated for various semiconductors (Si, Ge, and III-V materials) as well as complex oxides (perovskites, spinels, and garnets) (*2*–*4*, *6*–*8*). Successful use of graphitized silicon carbide (SiC) substrates for remote epitaxy and vdWE of GaN membranes has also been demonstrated recently (*4*, *8*). Single-crystalline SiC is one of the most promising candidates for remote epitaxy and vdWE due to its ability to form uniform defect-free and atomically flat wafer-scale graphene through high-temperature graphitization. The graphitization process eliminates the need to transfer graphene onto the host substrate for growth, preventing process-induced damage or residues on the graphene layer that can hinder the growth of high-quality epitaxial films. However, the graphitization and subsequent formation of graphene on SiC usually occur at very high temperatures over 1500°C (*9*, *10*). This high-temperature requirement is a technical obstacle as the high temperature not only causes pronounced step bunching of the SiC surface but also leads to nonuniform growth of graphene at the edges of the steps, generating multilayer graphene (MLG) stripes and unfavorable surface morphology for epitaxial growth (*9*).

Recently, several groups have reported on a graphitization method of SiC substrates using a thin metal film as a catalyst to reduce the graphitization temperature. This metal-assisted graphitization (MAG) process can be achieved through the deposition of various metals on the SiC substrate, such as nickel (Ni), iron (Fe), ruthenium (Ru), cobalt (Co), and its alloys (*11*–*25*). These studies showcase the catalytic effect of metals in breaking the Si─C covalent bond at temperatures below 1100°C. However, many groups have observed defective interface graphene at the metal silicide/SiC interface (*15*–*19*), suggesting that liberated carbon atoms diffuse inside the metal-silicide layer in the annealing process and then precipitate at the silicide/SiC interface to form graphene in the cooling process. Unfortunately, the silicide formation appears to be dominant (*13*, *16*), which not only deteriorates the uniformity and roughness of graphene (*13*, *18*) but also damage the SiC substrate surface (*15*, *17*). Notably, Lim *et al.* reported that SiC decomposes at ~450°C using Ni as the catalyst, much below the silicide formation temperatures, resulting in the formation of MLG (6~8 nm) at the metal/SiC interface due to the difference in the solubilities of carbon and silicon in Ni (*19*). However, the formation mechanism and properties of the interface graphene synthesized by MAG remain unclear due to limited information on surface morphology, domain size, and graphene uniformity as previous studies have predominantly focused on graphene formed on reacted metal surfaces, silicide surfaces, and the silicide/SiC interface. Thus, further investigation is needed to bridge this knowledge gap.

## RESULTS

**Fig. 1.**
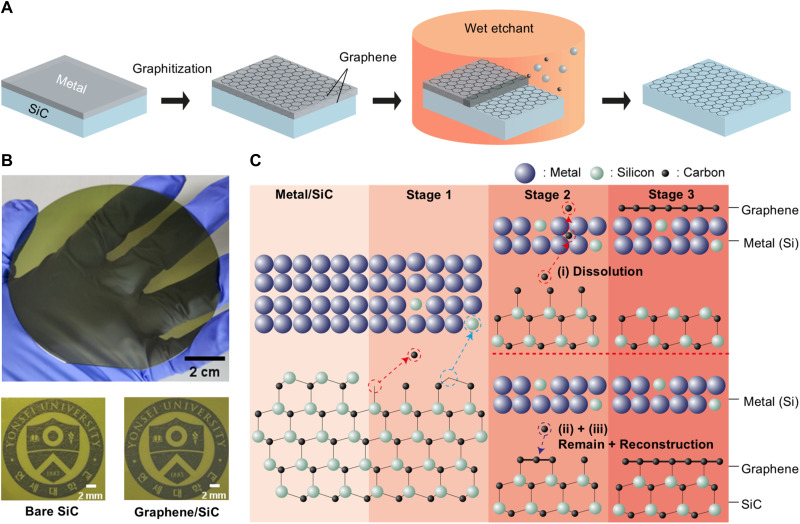
MAG process and carbon distribution model. (**A**) Schematic representation of the MAG process. A metal catalyst is deposited on a SiC substrate, annealed at a low temperature to form graphene, and subsequently removed using a wet etchant to yield a graphene/SiC platform. (**B**) Photograph of graphene on a 4-inch wafer SiC (left), with zoomed-in images showing bare SiC (top right) and MLG on MAG-treated SiC (bottom right). The graphene sample appears darker as a result of its reduced transmittance of visible light. (**C**) Schematic of the carbon distribution model during the solid-state reaction in the MAG process. The presence of graphene at the metal/SiC interface depends on the metal species, which governs the redistribution of carbon toward (i) dissolution into the metal bulk, (ii) retention at the interface to form a graphitic carbon layer, or (iii) reconstruction and recrystallization into pristine graphene. Black, light green, and dark blue spheres represent carbon (C), silicon (Si), and metal atoms, respectively.

A previous report using metal to reduce the graphitization temperature indicated that the metal film could act as a decomposition catalyst above a certain temperature, which is sufficient to fully overcome the activation barrier for silicide formation (*11*). The tendency of metal to react with Si reduces the Si─C bonding energy, thereby lowering the temperature required for Si sublimation and subsequent graphitization of the SiC. To get a complete understanding of this process, we carried out a meticulous study by varying the catalyst metal element and annealing temperature as well as performing density functional theory (DFT) calculations to fully understand the MAG process. The process of MAG is illustrated in Fig. 1A (see fig. S1 for the 4-inch wafer MAG process). A thin layer of metal is deposited onto a 4° off-axis 4H-SiC (0001) substrate using sputter deposition or e-beam evaporation for Ni, Ru, and Fe, followed by annealing in a rapid thermal annealing (RTA) chamber (see fig. S2 for temperature profile information). An off-axis orientation for the SiC wafer was selected over the on-axis substrate to promote periodic adatom nucleation at higher-density step edges, which enhances the nucleation for epitaxial growth and improves the crystallinity of the resulting film (*4*). After cooling, any residual metal film is etched away with its respective etchants. A photograph of the resulting metal-assisted graphitized SiC wafer is shown in Fig. 1B. The graphitized wafer shows a weak visible-light absorption, as indicated by the ultraviolet-visible (UV-vis) transmittance spectra (see fig. S3). Figure 1C illustrates the mechanism of how carbon atoms redistribute in the presence of metal catalysts at elevated temperatures, forming a graphene layer at the metal/SiC interface. The MAG process can be characterized into three distinct stages. In stage 1, once sufficient thermal energy is introduced, the metal catalyst facilitates the decomposition of Si─C bonds. In stage 2, as the annealing temperature increases, further dissociation occurs. The system then stabilizes as newly released C atoms either migrate outward into the metal bulk (i) or remain at the metal/SiC interface to form graphitic carbon or graphene layers (ii or iii). The redistribution of C atoms varies depending on the type of metal used due to differences in solubility and chemical affinity. Stable graphene layers are expected to form at the metal/SiC interface as a final product of the interactions (stage 3) when appropriate metals are used.

To understand the MAG processes illustrated in Fig. 1C, it is crucial to first examine the microscopic mechanism of MAG of SiC. Previous studies indicate that the dissociation of SiC is driven by its reaction with metals to form thermodynamically favored metal silicides (*26*–*28*). Ni, Ru, and Fe, which exhibit highly negative enthalpies of silicide formation (*29*), enable dissociation at lower temperatures compared to the conventional graphitization process. However, the distinct properties of each metal, such as the solubility of silicon and carbon in the metal and their chemical affinity with the metal, may strongly influence not only the distribution of silicon and carbon within the metal but also the interaction of the metal with SiC or graphene. As a result, substantially different behaviors during stages 2 and 3 are expected depending on the metal catalyst used, which has not been thoroughly investigated. In addition, most previous MAG studies primarily focused on carbon distribution after the metal had converted into silicide, where precipitated carbon was observed at both the silicide/SiC interface and the silicide surface due to the solubility limitations of carbon during the cooling stage. These approaches, however, overlooked the distribution of C and Si immediately after the Si─C bond dissociation in the initial stage, where the effect of the metal is assumed to be most critical. This lack of knowledge regarding the early stage of carbon redistribution in the MAG process has resulted in outputs unsuitable for device applications due to issues such as uneven SiC consumption (*16*) and graphene clustering (*13*), both attributed to localized silicide formation (*15*, *17*, *18*).

**Fig. 2.**
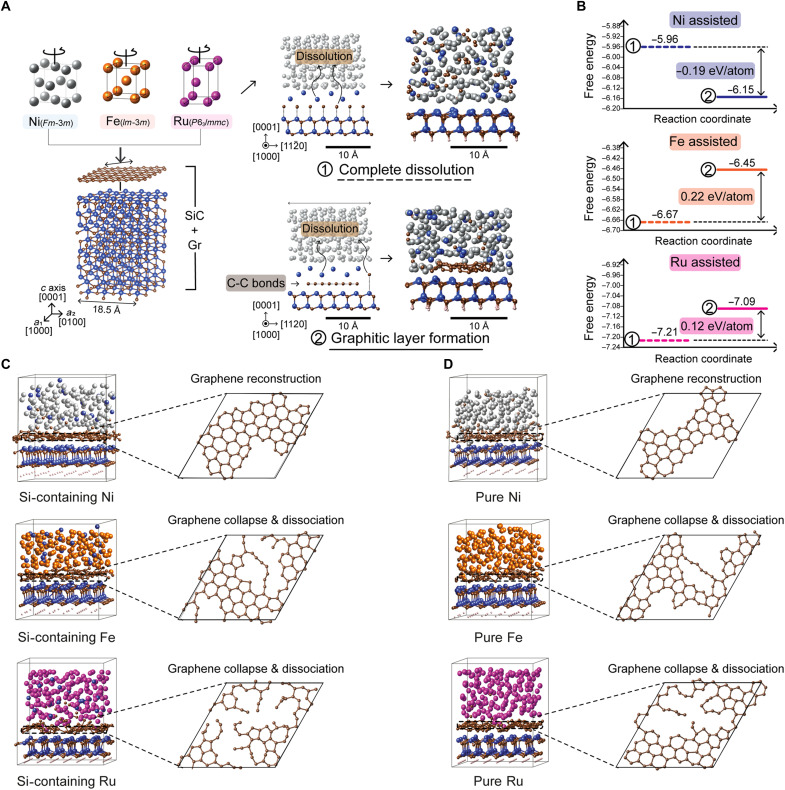
DFT modeling and atomic-scale simulation of carbon behavior at the metal/SiC interface. (**A**) Atomic-scale schematic of two representative pathways for carbon redistribution observed experimentally: (case 1) complete dissolution, in which carbon atoms released from the SiC substrate diffuse into the metal matrix, and (case 2) graphitic carbon formation, where carbon atoms remain at the metal/SiC interface and assemble into a graphene layer. (**B**) DFT-calculated formation energies for dissolution (case 1) and graphitization (case 2) at the Ni/SiC (top), Fe/SiC (middle), and Ru/SiC (bottom) interfaces. The results highlight the distinctive catalytic role of Ni in stabilizing interfacial graphene, attributed to its high silicon solubility and low carbon affinity. (**C** and **D**) Structural evolution of carbon at the metal/SiC interface during AIMD simulations for Si-containing (C) and Si-free metals (D). The trajectories of carbon atoms exhibit strong metal dependence: Ni promotes ordered graphene formation, whereas Fe and Ru induce structural disorder and collapse of the graphene layer.

To bridge the gap in understanding the microscopic processes at the early stage of the MAG process, as well as to elucidate the metal-specific differences, we used ab initio molecular dynamics (AIMD) simulations. We considered three metal elements—Ni, Ru, and Fe—as catalysts and investigated the initial stage of carbon redistribution following Si─C bond dissociation to unveil the dependence of stable graphene formation on metal types (see Supplementary Note 1 and figs. S4 and S5 for detailed simulation methods). As illustrated in Fig. 2A, two competing pathways for carbon behavior were identified: complete dissolution (case 1), where C atoms released from the SiC substrate diffuse directly into the metal matrix, and graphitic carbon formation (case 2), where carbon atoms remain at the metal/SiC interface and form a graphene layer. As shown in Fig. 2B, the comparative formation-energy analyses reveal that graphene formation at the metal/SiC interface is energetically favorable only for Si-dissolved Ni, whereas Fe and Ru exhibit positive formation energies, indicating that these metals prefer carbon dissolution over stabilizing a graphene layer. These findings indicate the distinctive catalytic effectiveness of Ni in promoting stable interfacial graphene formation. The structural evolution observed during AIMD simulations (fig. S6) supports these trends. Interesting observations include (i) carbon and metal atoms tend to diffuse into each other to a certain extent at the metal/SiC interface, resulting in slight intermixing between the carbon and metal elements (stage 2); (ii) carbon atoms in the graphene layer maintain their hexagonal arrangement, with the degree of structural order depending on the metal type; and (iii) the van der Waals gap between the metal and graphene layer is relatively well maintained in the Ni-containing model (stage 3), whereas Fe- or Ru-containing models show a collapse of the gap (fig. S7). To evaluate whether graphene can remain structurally stable under Si-free conditions during the initial stage, we compared both initial (Si-free) and Si-containing models (figs. S6 and S8). As shown in Fig. 2 (C and D), both Si-containing and pure Ni models preserve the hexagonal graphene structure, indicating effective stabilization at the metal/SiC interface during stage 2 (see movie S1 for atomic trajectories from AIMD simulations and fig. S9 for a detailed analysis of the structural evolution). In contrast, Fe- and Ru-containing models exhibit pronounced graphene collapse and dissociation (movies S2 and S3, respectively). These AIMD results highlight the distinctive catalytic role of the Si-dissolved Ni matrix in driving carbon reorganization and graphene-like structure formation. Ni, known for its high silicon solubility (*30*) and low carbon affinity (*26*), facilitates graphene formation at the metal/SiC interface. In contrast, graphene layers become unstable with Ru [low Si solubility (*31*)] and Fe [high carbon affinity (*32*)], emphasizing the importance of selecting a catalytic metal with high Si solubility and low carbon chemical affinity.

**Fig. 3.**
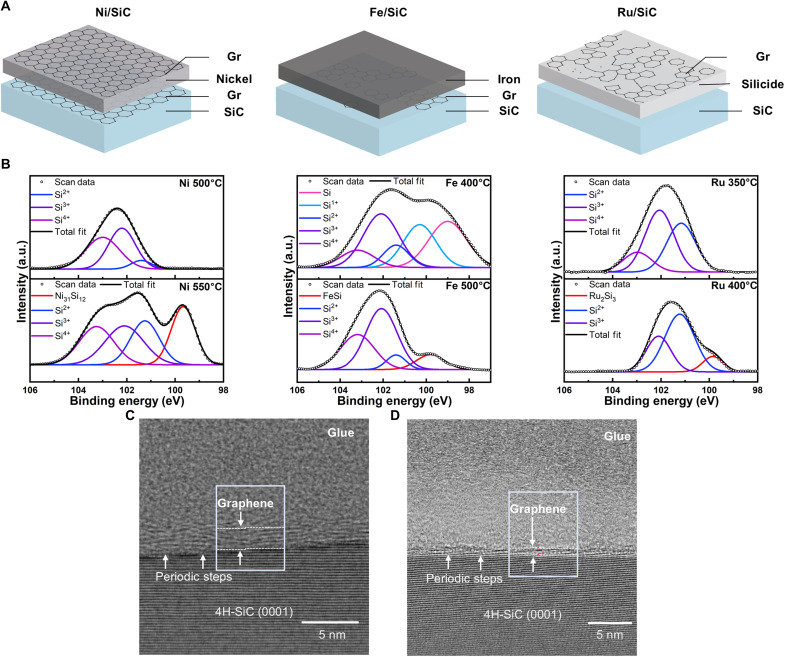
Metal effect in the MAG process and experimental investigations. (**A**) Schematic representation of the metal effect in the MAG process, based on DFT calculations and experimental results. A continuous graphene layer at the metal/SiC interface was obtained only in samples annealed with Ni as a catalyst without silicide reaction. Gr, graphene. (**B**) XPS spectra of the Si 2p core level for various metal/SiC systems after annealing. Detection of liberated Si atoms within the metal bulk for Ni, Fe, and Ru confirms that all three catalysts promote Si─C bond dissociation at low temperatures. a.u., arbitrary units. (**C** and **D**) High-resolution cross-sectional TEM images of the MAG-treated SiC surface after Ni removal. (C) Deposition of the 50-nm Ni layer yields MLG on SiC. (D) Reducing the Ni layer thickness to 8 nm produces thinner FLG on SiC, demonstrating control over the number of graphene layers. This reduction is attributed to shorter diffusion pathways and a diminished driving force for Si─C dissociation in the thinner metal films.

These DFT results align well with our experimental results, demonstrating that only Ni enables the formation of uniform graphene at the metal/SiC interface without any silicide formation. A schematic representation of the metal’s effect on MAG, highlighting the pronounced differences in outcomes, is illustrated in Fig. 3A. To experimentally verify the catalyst effect of each metal in reducing the Si─C dissociation barrier energy during the MAG process, we performed x-ray photoelectron spectroscopy (XPS) measurements of the Si 2p and C 1s core levels after annealing at remarkably low temperatures. As shown in Fig. 3B, Si 2p XPS spectra confirmed the liberation of Si atoms when annealing the samples at 500°C for Ni and Fe and at 400°C for Ru, showcasing a substantial reduction in the energy required for Si─C bond dissociation. The formation of carbon layers on the metal surface, produced either by dissociated carbon diffusing through grain boundaries (*21*) or due to the low solubility of carbon in the metal matrix (*20*), was further confirmed by C 1s core-level XPS and Raman spectroscopy (figs. S10 and S11).

After confirming Si─C bond dissociation in all metal/SiC systems, we investigated the temperature-dependent phase transformations in the metals, including the formation of silicide through solid-state reactions. These transformations were characterized using Si 2p XPS and x-ray diffraction (XRD) measurements (fig. S12). Notably, no silicide peaks were detected for Ni annealed at 500°C or Fe annealed at 400°C, despite clear evidence of liberated Si atoms observed in the Si 2p spectra. However, at higher temperatures, peaks at 99.6 eV for Ni and 99.7 eV for Fe were observed, corresponding to the formation of Ni_2_Si and Ni_31_Si_12_ mixed phases (*33*) and FeSi (*34*), respectively. Ru, in contrast, exhibited a distinct peak at 99.8 eV, indicative of Ru_2_Si_3_ formation (*35*) upon annealing at 400°C. However, the XRD analysis revealed the presence of the Ru_2_Si_3_ phase even at 350°C, suggesting that silicide formation occurred beneath the typical XPS probing depth (~10 nm). Additional confirmation of structural changes was provided by metal core-level XPS, oxide (O) core-level XPS, and Raman spectroscopy (figs. S10 and S13). We further observed distinct effects of each metal on graphene formed at the metal surface. A distinct 2D peak in Raman spectra was only detected on Ni without the formation of a silicide layer (fig. S14). For Fe, graphene formation occurred only after silicide formation, where liberated carbon atoms were either contained within the metal or distributed at the metal/SiC interface. In contrast, Ru failed to form a uniform graphene film, instead producing an amorphous-like graphitic layer below 400°C, despite the silicide reaction having already proceeded, potentially indicating a need for higher temperatures to crystallize this graphitic layer into graphene (*11*, *16*).

Because DFT calculations indicated substantial differences in the possibility of graphene formation at the interface of each metal/SiC depending on the metal, further investigations of the metal/SiC interface were conducted. The residual metal film was etched away using its respective etchant after annealing at the minimum temperature needed for Si─C dissociation. Raman spectra and the optical image [optical microscopy (OM)] confirmed that a graphene layer was synthesized on SiC only when Ni was used as the catalyst, in agreement with DFT results (fig. S14). In contrast, no carbon layer was detected for Ru annealed at 350°C, whereas an uneven poorly crystallized graphitic layer was observed for Fe annealed at 400°C, suggesting that the carbon at the metal/SiC interface either diffused outward into the reacted region or was absorbed into the metal layer, again consistent with the DFT results. These results unambiguously verify that the choice of the metal catalyst plays a crucial role in the MAG process. Among the three metals studied, only Ni was able to form wafer-scale graphene at the metal/SiC interface, without any silicide or carbide formation.

After selecting the appropriate metal (Ni) on the basis of the above results, further experimental studies were conducted to validate the carbon distribution model at both low temperatures (stage 1 in Fig. 1C) and relatively high temperatures (stage 2 in Fig. 1C). We further lowered the annealing temperature not only to control the thickness of the graphene but also to explore the relationship between annealing temperature and Si─C bond dissociation. As shown in Fig. 3C and fig. S15, transmission electron microscopy (TEM) analysis confirmed the formation of MLG (>2 graphene monolayers) at the Ni/SiC interface after annealing at 500°C for ~1 s, whereas few-layer graphene (FLG; ≤2 graphene monolayers) formed at 320°C for ~1 s (with the thickness criteria distinguishing FLG and MLG defined in fig. S3). These results are consistent with the UV-vis transmittance spectra obtained at the macroscopic scale after etching with the respective etchant (fig. S16). Comparable trends were observed in electron beam–deposited Ni experiments and under different ramp speeds (figs. S16 and S17). Furthermore, when the annealing temperature was increased to 550°C, we observed not only inhomogeneous silicide reactions but also clustering of graphene, as confirmed by atomic force microscopy (AFM), scanning electron microscopy (SEM), and Raman spectroscopy (fig. S18). These findings align with observations reported in the literature (*15*, *20*).

Furthermore, we reduced the thickness of Ni from 50 to 8 nm to investigate the relationship between metal thickness and graphene thickness. As dissociated Si atoms are homogeneously distributed within the Ni lattice (*36*), we hypothesized that reducing the metal thickness would decrease the reactivity between SiC and Ni. This reduction is attributed to the lower gradient-driven diffusion of Si atoms at thinner metal layers, resulting from shorter diffusion pathways or a diminished driving force for Si─C dissociation, thereby leading to thinner graphene on the SiC surface. As shown in Fig. 3D, the thickness of graphene on the SiC surface decreased to FLG. Once again, the UV-vis transmittance confirmed decreasing transmittance due to the increased graphene thickness as the metal thickness increased (see fig. S19). Overall, the results indicate that the MAG process is primarily temperature driven, but the thickness of the graphene layer can be controlled by adjusting the metal layer thickness or the annealing temperature.

**Fig. 4.**
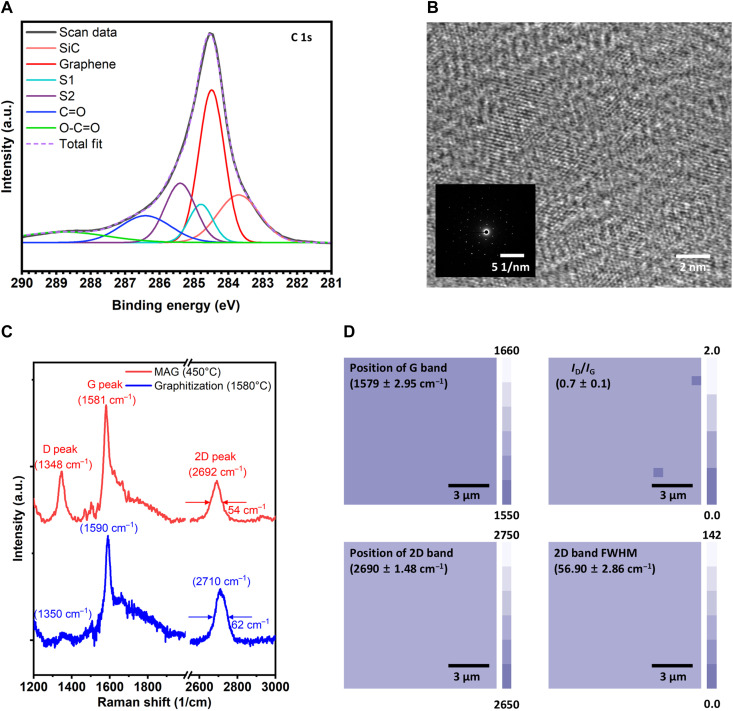
Wafer-scale single-crystalline graphene formed on SiC via the MAG process. (**A**) XPS spectra of the C 1s region, showing graphene on the entire SiC. (**B**) Plan-view TEM image with SAED patterns of graphene transferred onto a TEM grid, verifying the single-crystalline nature of the graphene. (**C**) Raman spectra displaying clear D, G, and 2D peaks, confirming the presence of graphene on SiC, with quality comparable to that of graphene synthesized via high-temperature graphitization. (**D**) Raman maps of the G and 2D peak positions, *I*_D_/*I*_G_ ratio, and 2D FWHM, demonstrating the uniformity of graphene quality and thickness. A total of 256 points were measured within a 15 μm–by–15 μm Raman mapping area.

Next, we characterized the MLG formed on SiC by etching away the Ni layer graphitized at 500°C for ~1 s. The presence of graphene was confirmed by XPS, Raman spectroscopy, and plan-view TEM. As shown in Fig. 4A, the C 1s spectrum consists of a SiC bulk component at 283.7 eV, the sp^2^ C═C bond of graphene at 284.5 eV, and two surface buffer layer components, S1 (284.8 eV) and S2 (285.4 eV). The S1 and S2 peaks exhibited an intensity ratio of ~1:2, with full width at half maximum (FWHM) values of 0.8 and 1.0 eV, respectively. These peaks may originate from the graphene buffer layer (GBL); S1 corresponds to carbon atoms partially bonded to underlying Si atoms, whereas S2 is attributed to sp^2^-bonded carbon exhibiting stronger in-plane bonding within the buffer layer (*37*, *38*). Such peaks are characteristic of epitaxially grown graphene on SiC and are observed in both the MLG/SiC and the FLG/SiC samples, suggesting the presence of a GBL in each case (see fig. S20). We note, however, that the buffer layer is not directly discernible in TEM images (Fig. 3, C and D, and fig. S30), likely due to contrast limitations arising from the dense step-terrace structure of the SiC substrate. In addition, the XPS Ni 2p spectra and energy-dispersive x-ray spectroscopy confirmed the complete removal of Ni as it was not detected on the surface (fig. S21). The graphene was also characterized using plan-view TEM (see Materials and Methods). The TEM image and selected-area electron diffraction (SAED) pattern reveals a well-aligned honeycomb lattice, providing clear evidence of single-crystalline dominant graphene, as shown in Fig. 4B.

To evaluate the characteristics of graphene and compare them with the high-temperature graphitized sample on a macroscopic scale, Raman spectroscopy was performed. As shown in Fig. 4C, both spectra confirmed the formation of graphene, as evidenced by the characteristic D (~1350 cm^−1^), G (~1580 cm^−1^), and 2D (~2700 cm^−1^) bands (*39*). The MAG-treated graphitized sample exhibited a more pronounced D peak (*I*_D_/*I*_G_ ≈ 0.5) compared to the high-temperature graphitized sample (*I*_D_/*I*_G_ ≈ 0.1), indicating a higher density of structural imperfections, which can include grain boundaries, point defects, surface corrugations, or substrate-induced perturbations (*40*). However, the clear SAED pattern confirmed the preservation of single-crystalline order, ruling out grain boundaries as a major contributor to the D band. Instead, the enhanced D band is attributed to point defects, such as vacancies or sp^3^-type defects (*41*, *42*). Both samples exhibited distinct G and 2D bands, commonly used as indicators of crystalline quality and thickness of graphene (*39*–*45*). The positions of the G and 2D bands of the MAG-treated SiC remained nearly unchanged compared to pristine graphene, indicating negligible doping and strain effects (*40*, *43*). These results suggest that MAG-treated SiC effectively preserves the structural quality of graphene without inducing noticeable lattice perturbations. In contrast, the high-temperature graphitized sample exhibited a marked upshift in both the G band (~10 cm^−1^) and the 2D band (~30 cm^−1^), indicating considerable lattice distortion driven by charge transfer or residual strain. The FWHM of the 2D band serves as a quantitative measure not only of graphene thickness, ranging from single-layer graphene (typically 27.5 ± 3.8 cm^−1^) to four-layer graphene (typically 63.1 ± 1.6 cm^−1^) (*9*, *46*), but also of the long-range crystalline order (*42*, *47*). The narrower 2D band FWHM observed in the MAG-treated graphene relative to the high-temperature counterpart suggests similar crystalline quality at comparable thicknesses. As shown in Fig. 4D, Raman mapping revealed highly uniform G and 2D positions (1579 ± 2.95 and 2690 ± 1.48 cm^−1^, respectively). The 2D band also exhibited a consistent FWHM (averaging 56.90 ± 2.86 cm^−1^) across the substrate. This spatial uniformity provides strong evidence for the high structural homogeneity and consistent thickness of the graphene film across the entire SiC wafer. Furthermore, complete graphene coverage across the 4-inch SiC substrate was confirmed (see Raman spectra and camera image in fig. S22), demonstrating the capability to produce 4-inch graphene directly on a semiconductor substrate using a very low-temperature, ultrafast process that relies solely on RTA and sputtering, which are techniques fully compatible with Si CMOS (complementary metal-oxide semiconductor) industry standards.

The results of MAG suggest that the SiC substrate can be used as an ideal substrate for producing single-crystalline freestanding high-quality III-nitride films by remote epitaxy and vdWE. Remote epitaxy is an advanced epitaxial technique where epitaxial growth occurs on a substrate covered with a 2D material (*1*). This process typically occurs on few-layer 2D materials, which are thin enough to allow partial penetration of the electrostatic potential from the underlying substrate (*1*–*3*, *48*). At the substrate-epitaxial membrane interface, the 2D material and its van der Waals gap effectively eliminate dislocations or cracks in the epitaxial membrane caused by lattice strain relaxation, which has been a persistent challenge in conventional techniques for achieving high-quality epitaxial devices (*2*, *49*, *50*). In contrast, vdWE involves epitaxial growth on multilayer 2D materials, which is thick enough to completely screen the substrate potential. This creates a slippery van der Waals interface that enable strain relaxation, allowing the growth of materials with substantial lattice mismatches greater than 60% (*1*). This mechanism is further illustrated and discussed in fig. S23. Furthermore, in both 2D-based epitaxial techniques, epitaxial membranes are freestanding on the substrate and only bound to the substrate via a weak van der Waals force. This characteristic enables the fabrication of freestanding membranes through 2D material–assisted layer transfer (2DLT) (*7*). These approaches are particularly advantageous as they allow the repeated reuse of expensive substrates while producing multiple single-crystalline membranes. A schematic of the detailed 2D-based epitaxy and 2DLT process is shown in fig. S24.

A key advantage of our MAG platform is the ability to precisely tune the graphene thickness, enabling the investigation of both remote epitaxy (on FLG) and vdWE (on MLG) on a single substrate system. To demonstrate the applicability of our MAG-treated SiC substrate, III-nitrides films were epitaxially grown. III-nitride materials such as GaN and AlN are promising candidates for power and radio frequency devices as well as light-emitting diodes due to their exceptional intrinsic material properties (*51*, *52*). These include wide direct bandgaps (3.4 eV for GaN and 6.2 eV for AlN) (*52*, *53*), high breakdown electric field (4.9 MV cm^−1^ for GaN and 15.4 MV cm^−1^ for AlN) (*54*), and high electron mobility. These materials are particularly well suited for 2D-based epitaxial growth on graphitized SiC as their hexagonal lattice arrangement aligns well with SiC compared to other available substrates, allowing high-quality single-crystalline growth via remote epitaxy. Furthermore, the graphitization of SiC produces nearly pristine graphene, whose preserved hexagonal lattice structure enhances the growth of *c*-plane III-nitrides films, offering substantial benefits for vdWE (*50*). Last, the direct synthesis of graphene on SiC eliminates the need for wet-transferred graphene synthesized on metal foils via chemical vapor deposition (CVD), which often introduces defects such as wrinkles, holes, interfacial contamination, and organic residues. These defects can disrupt the remote interaction between the substrate and the remote epitaxial film, as well as between the graphene and the van der Waals epitaxial film (*2*).

**Fig. 5.**
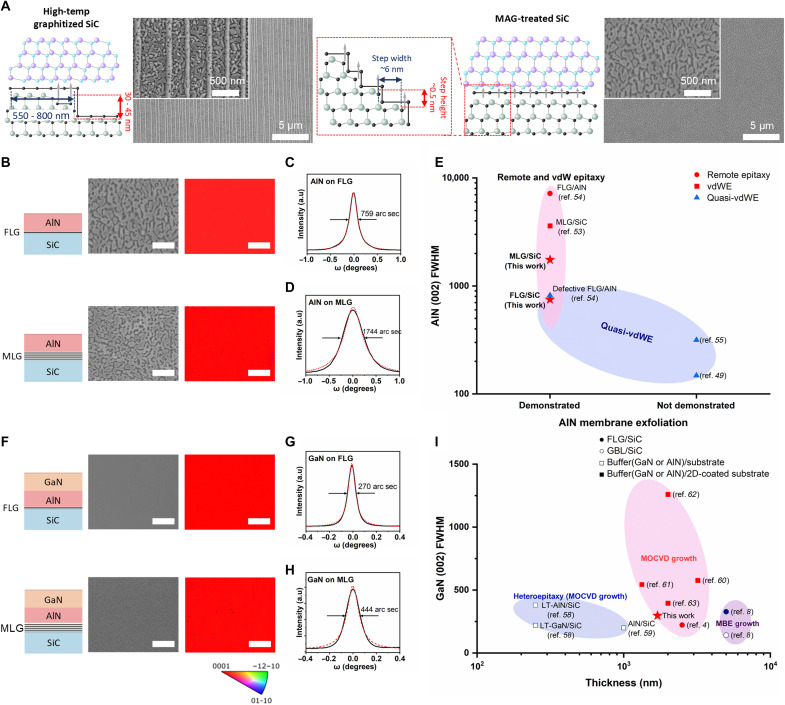
Freestanding III-nitride membranes synthesized on MAG-treated graphene/SiC templates. (**A**) Schematic illustration and SEM images of 2D-assisted epitaxy on MAG-treated graphitized SiC and high-temperature graphitized SiC. Unlike high-temperature graphitization, MAG-treated SiC avoids step bunching, offering potential advantages for growing high-quality membranes through 2D-assisted epitaxy. (**B**) Schematic illustration of the grown film structure (left), SEM image (middle), and EBSD maps (right), for AlN membranes on FLG/SiC and MLG/SiC templates. SEM images show regions that appear open and not fully coalesced, whereas EBSD maps, acquired under the ~70° tilt geometry, primarily reflect the orientation of the exposed AlN facets and therefore exhibit a uniform *c*-axis contrast over a large scale. (**C** and **D**) XRD ω-scan of both templates. The FWHM of the AlN (002) peak increases with graphene layer thickness. (**E**) XRD benchmark of the exfoliated AlN membrane, showing the lowest (002) FWHM, as referenced in the main text. (**F**) Schematic illustration of the grown film structure (left), SEM image (middle), and EBSD map (right), showing the highly single-crystalline nature of the GaN membrane over a large scale on the AlN/FLG/SiC template. (**G** and **H**) XRD ω-scans of GaN membranes grown on AlN/FLG/SiC and AlN/MLG/SiC templates, respectively, showing no substantial differences. (**I**) XRD FWHM benchmark of the exfoliated GaN membrane, confirming the high crystalline quality achieved. The scale bars in all SEM images and EBSD maps are 1 μm.

Although the advantages of the graphitized SiC template for 2D-based epitaxy are clear, high-quality III-N membrane growth on graphene faces challenges due to its low chemical reactivity (*55*). The high surface migration rate of group III metals on slippery graphene prevents nuclei from stabilizing, which results in highly defective epitaxial films due to insufficient nucleation sites (*50*). High-quality single-crystalline AlN film growth via quasi-vdWE on graphitized SiC has been reported, wherein plasma treatment was applied to enhance nucleation by introducing defects on the graphene surface (*50*, *56*, *57*). Although partial exfoliation of AlN layers synthesized through quasi-vdWE has been demonstrated (*56*), full-area transfer remains challenging due to the strengthened interfacial bonding between the AlN epilayer and the defect-modified graphene. As a result, previous studies have primarily focused on synthesizing crack-free AlN layers that leverage the stress relaxation benefits of graphene. Because the membrane exfoliation yield improves with a uniform graphene layer covering the entire surface on the substrate, untreated graphene is required to successfully produce freestanding membranes. The successful exfoliation of GaN on nondefect-induced graphitized SiC via 2D-assisted epitaxy was first reported in 2014, achieved not only by engineering the epitaxial growth strategy on a 2D surface but also by using the periodic step edges of SiC (*4*). These step edges, with terrace widths typically ranging from 550 to 800 nm and step heights from 30 to 45 nm, remain after the step bunching induced by high-temperature processes (*58*). These periodic step edges generate uniform fluctuations in electric potential, providing energetically favorable nucleation sites for adatoms and enabling the growth of single-crystalline GaN (*4*). In this regard, our MAG-graphitized approach is expected to offer considerable advantages. The MAG process is conducted at low temperatures, which avoids step bunching and preserves the naturally periodic nanoscale terraces of 4° offcut 4H-SiC (see AFM images and line profile in fig. S25). These terraces exhibit widths below 7.2 nm and step heights under 0.5 nm (*59*). To verify this advantage, we grew AlN on both high-temperature graphitized and MAG SiC substrates. As illustrated in Fig. 5A, growth on high-temperature graphitized SiC resulted in very rough nonuniform polycrystalline AlN growth following the step-bunched surface morphology (see in-plane crystallinity in fig. S26), whereas the MAG SiC substrate resulted in smooth single-crystalline AlN growth.

High-quality AlN films were grown on both MAG-treated MLG/SiC substrate and FLG/SiC substrate by vdWE and remote epitaxy, respectively. For both samples, electron backscatter diffraction (EBSD) maps with SEM images and XRD scans confirmed a (002) wurtzite orientation across a large area, as shown in Fig. 5 (B to D) (see in-plane crystallinity in fig. S27). These results indicate that single-crystalline AlN can be grown via remote epitaxial seeding from FLG/SiC and van der Waals epitaxial seeding from MLG/SiC. The FWHM of AlN (002) peaks in XRD scans broadened with increasing graphene thickness on SiC, suggesting lower seeding efficiency compared to remote epitaxy. Prior studies reported on the successful exfoliation of high-quality AlN on graphitized SiC grown via 2D-based epitaxy, achieving an AlN (002) FWHM of 3600 arc sec (*55*) and partial exfoliation of high-quality AlN via quasi-vdWE, with a (002) FWHM of 792 arc sec and (102) FWHM of 1044 arc sec (*56*). In contrast, our study demonstrated markedly improved AlN quality on MAG-treated SiC, with remote epitaxy achieving a (002) FWHM of 759 arc sec and a (102) FWHM of 833 arc sec, whereas vdWE-grown AlN exhibited broader peaks, with a (002) FWHM of 1744 arc sec and a (102) FWHM of 2136 arc sec, at a film thickness below 300 nm (see fig. S28). The exfoliated AlN layer exhibiting the lowest (002) FWHM is presented in Fig. 5E, highlighting the high crystalline quality achieved through our approach. We concluded that the markedly enhanced crystallinity of AlN can be attributed to the presence of tightly packed and periodically stepped SiC. These features provide highly energetically favorable nucleation sites, which are effective for both remote epitaxy (*4*) and vdWE (*50*, *57*).

After confirming the successful growth of a single-crystalline AlN layer on MAG-treated SiC, we used it as a buffer layer to grow single-crystalline GaN films. In conventional epitaxy, AlN layers (*a* axis: 3.112 Å) are commonly used as intermediate layers to address the lattice mismatch between GaN (*a* axis: 3.189 Å) and SiC (*a* axis: 3.073 Å), thereby enhancing the quality of GaN. Although graphene as a buffer layer effectively relaxes the lattice strain of GaN thin films, the crystallinity of GaN grown on AlN synthesized via 2D-assisted epitaxy still requires thorough investigation. To evaluate this, we grew GaN on each 2D-assisted epitaxial AlN templates and conducted XRD and EBSD measurements to determine the crystallinity on a macroscopic scale. The EBSD maps with SEM images and XRD scans verified the (002) wurtzite orientation over a large area, indicating single crystallinity of the grown GaN film on both AlN templates, as shown in Fig. 5 (F to H) (see in-plane crystallinity in fig. S29). Notably, the FWHM values of GaN (002) were 270 and 444 arc sec for remote epitaxy and vdWE, respectively, whereas the GaN (102) FWHM values were 509 and 760 arc sec for remote epitaxy and vdWE, respectively. These results are comparable not only to those of AlN buffer–assisted GaN films on conventional SiC via metal-organic chemical vapor deposition (MOCVD) (*60*, *61*) but also to the remote epitaxial growth of GaN on graphitized SiC and other 2D-coated substrates (*4*, *8*, *62*–*65*), as shown in Fig. 5I. The crystal quality can be further improved by optimizing the growth recipe. After confirming the single crystallinity of the GaN layer, cross-sectional TEM analysis further verified that the graphene interlayers remain intact after GaN/AlN growth (fig. S30). We then used 2DLT to exfoliate both samples. Following the deposition of the adhesion layer (Ti) and stressor layer (Ni) on the surface of the GaN samples, mechanical exfoliation was carried out using thermal release tape (TRT) as a handling layer. The strain energy generated by the Ni stressor guided crack propagation precisely along the AlN/graphene interface, facilitated by the weak van der Waals bonds between the GaN/AlN and the graphitized SiC. As a result, freestanding GaN/AlN membranes were successfully obtained for both MAG-treated templates, and the underlying graphene/SiC templates remained largely intact after exfoliation (see figs. S31 and S32).

## DISCUSSION

In conclusion, we demonstrate low-temperature ultrafast growth of wafer-scale graphene on a 4-inch SiC substrate below 500°C, with precise control on the number of graphene layers by changing the metal thickness and annealing temperature. The resulting graphene grown by MAG exhibits overall uniform characteristics comparable to graphene synthesized by traditional high-temperature graphitization. Moreover, the III-nitride films grown on MAG-treated SiC via 2D-assisted epitaxy demonstrate quality comparable to those grown on high-temperature graphitized SiC and conventional substrates such as sapphire and SiC by MOCVD. We found that the metal used strongly influences the presence of graphene layers on SiC, with Ni being the only catalyst capable of synthesizing uniform graphene on SiC. These results offer valuable insights into the general mechanisms of the MAG process, providing a more accessible approach for synthesizing wafer-scale graphene, which can be readily used for remote epitaxy or vdWE, as well as produce stripe-free single-crystalline graphene at wafer-scale. Our approach not only markedly reduces the barrier of preparing epitaxial graphene coated single-crystalline substrates but also substantially enhances the crystallinity of the freestanding single-crystalline membranes for heterogeneous integration.

## MATERIALS AND METHODS

### Computational details

The first-principles calculations were performed using the projected augmented wave (PAW) plane-wave basis, implemented in the Vienna ab initio simulation package (VASP) (*66*). An energy cutoff of 520 eV was used, and the atomic positions were optimized using the conjugate gradient scheme without any symmetric restrictions, until the maximum force on each of them was less than 0.01 eV/Å (*67*). All atoms were relaxed to their equilibrium positions when the change in energy on each atom between successive steps converged to 1 × 10^−6^ eV/atom. The heterostructure was modeled with an 8 by 8 by 1 grid for k-point sampling. The generalized gradient approximation (GGA) exchange-correlation (XC) DFT functional Perdew-Burke-Ernzerhof (PBE) was used for geometrical optimization and electronic structure calculations (*68*). The slab models had dangling bonds on the vacuum surface terminated by pseudo-hydrogen atoms with appropriate fractional charges to avoid surface states. To determine the vacuum level, dipole corrections are introduced to compensate for the artificial dipole moment at the open ends (20-Å vacuum space along the *c* axis) arising from the periodical boundary condition imposed in these calculations (*69*). AIMD simulations were performed in supercells using DFT calculations with a gamma-centered k-point. The time step was set to 3 fs. Simulations for 3 ps were run with a time step of 3 fs to study the dynamic graphitization process. The temperature of the simulation system was controlled at 2273 K using the Nosé-Hoover thermostat (*70*, *71*).

### Sample preparation

The single-crystalline 4° offcut 4H–SiC (0001) substrates were supplied by Cree, Inc. To remove organic contaminants, substrates were cleaned sequentially for 5 min in acetone, 5 min in isopropyl alcohol (IPA) in an ultrasound bath, and lastly dried by a nitrogen gun.

### Metal deposition and graphene formation/transfer method

After preparing the SiC substrate, we deposited Ni, Fe, and Ru onto each SiC substrate to investigate the effect of metal catalysts on the interface graphene layer. The primary method for Ni deposition was dc magnetron sputtering using an apparatus with an Ar plasma at room temperature (JURA deposition apparatus made by Vakuum Servis Ltd., Czech Republic). The sputtering was carried out in an Ar atmosphere (pressure: 0.5 mtorr) with a dc power of 100 W. The resulting deposition rate was 2.5 nm/min. Fe and Ru were deposited on the substrate using an e-beam evaporator (Korea Vacuum Tech., Korea Republic). The base pressure was maintained below 5 × 10^−7^ torr, with a deposition rate of 0.6 Å/s. For the comparative study of deposition methods shown in fig. S16, Ni was also deposited using this e-beam evaporation method.

Following metal deposition, the samples underwent RTA. The base pressure during RTA was maintained at 7 × 10^−3^ torr, with 1 torr of N_2_ added to prevent metal oxidation. The samples were annealed for 3 min to determine the temperature required to decompose the Si─C bond and assess whether it induces a phase change of metals into silicide. XPS (K-alpha, Thermo Fisher Scientific Inc.) using an aluminum anode (spot size: 400 μm; energy resolution: FWHM ≤ 0.5 eV for Ag 3d_5/2_; pass energy: 40 eV; step size: 0.1 eV) was used to analyze the elemental diffusion and chemical bonding at the surface. In parallel, XRD (Rigaku SmartLab, Cu k_α1_, λ = 1.54051 Å) was used to identify crystalline phases and monitor structural transformations within the metal/SiC structure. Raman spectroscopy (Horiba Jobin Yvon, LabRam Aramis) equipped with a 532-nm wavelength laser was used to analyze phase transformations of metals and to confirm the presence of graphene. Following the reaction, the interfacial graphene layer was analyzed using high-resolution transmission electron microscopy (JEOL ARM200F). To further investigate the interface characteristics, the samples were immersed in a 40% (w/v) ferric chloride (FeCl_3_) solution (for Ni and Fe) for 5 min or a 5% (w/v) sodium hypochlorite (NaOCl) solution (for Ru) for 5 min. The samples were then characterized using OM, SEM (JEOL, JSM-IT-500HR), UV–vis–near-infrared spectroscopy (JASCO, V-650), AFM (Park Systems, NX-10), TEM, and Raman spectroscopy. Raman analysis was performed after normalizing all spectra and subtracting the signal from bare SiC (specifically the SiC band at 1520 cm^−1^, which exhibits the maximum intensity across all samples within the spectral range of 1200 to 3000 cm^−1^) to enable precise analysis of the graphene signal.

After graphene synthesis, polyvinyl alcohol (PVA) was drop-casted onto the surface and baked at 80°C for 5 min to form an adhesion layer. A TRT was then attached, facilitating the detachment of graphene through mechanical exfoliation. The exfoliated graphene was transferred onto a TEM grid. The TRT was removed by baking the sample at 130°C, and the graphene was revealed by dipping the sample into deionized water.

### SiC high-temperature graphitization

The wafer was loaded into an Aixtron VP508 reactor for graphitization. It was first cleaned in a hydrogen environment for 30 min at 1520°C, followed by annealing at 1580°C in a 700-torr argon ambient for 10 min.

### III-N epitaxial growth and exfoliation

The GaN/AlN heterostructure was epitaxially grown on a graphene/SiC substrate by an MOCVD reactor equipped with a vertical showerhead-type chamber from Sysnex Co. Ltd. The MOCVD reactor maintained a stable pressure of 30 torr with hydrogen as a carrier gas throughout the growth process. Initially, the AlN buffer layer was grown at 1050°C for 40 min on FLG/SiC and MLG/SiC. Subsequently, the GaN layer was grown by a single-step growth at 1100°C for 10 min on each AlN layer. In this growth process, trimethylgallium, trimethylaluminum, and ammonia were used as the sources of gallium, aluminum, and nitrogen, respectively. The resulting GaN/AlN epilayers were exfoliated using a Ti/Ni stressor stack with a handling layer. A 50-nm thick Ti layer was deposited as an adhesion layer for the Ni stressor layer via e-beam evaporation, followed by the deposition of a 3.5-μm-thick Ni stressor layer using dc magnetron sputtering under an argon ambient. After the deposition of Ti/Ni layers, the TRT was attached as a handling layer. Last, the TRT/Ti/Ni/epi stack was lifted from the edges, enabling precise and controlled exfoliation of the GaN/AlN epilayers.

### III-N membrane characterizations

The plan-view, cross-sectional, and EBSD images of the grown and exfoliated samples were obtained using a field-emission SEM system (SU8220, Hitachi). XRD characterization was carried out using an XRD system (Empyrean, Malvern Panalytical, Cu K_α1_, λ = 1.54051 Å) to identify crystalline phases and assess structural evolution.

## Supplementary Materials

### The PDF file includes:

Supplementary Note S1 Figs. S1 to S32 Tables S1 and S2 Legends for movies S1 to S3 References
