# Supplementary material for: A universal 2D-on-SiC platform for heterogeneous integration of epitaxial III-N membranes
Source: Sci Adv. 2025 Nov 21;11(47):eadz3605. doi: 10.1126/sciadv.adz3605 (PMC12637313; doi:10.1126/sciadv.adz3605)
Supplement: Supplementary file 1 — Supplementary Note S1 Figs. S1 to S32 Tables S1 and S2 Legends for movies S1 to S3 References [file sciadv.adz3605_sm.pdf]

Science Advances
AAAS

Supplementary Materials for

A universal 2D-on-SiC platform for heterogeneous integration of epitaxial III-N membranes

Se H. Kim *et al.*

Corresponding author: Hongsik Park, hpark@ee.knu.ac.kr; Sungkyu Kim, sungkyu@sejong.ac.kr; Tae Hoon Lee, thl@knu.ac.kr; Hyun S. Kum, hkum@yonsei.ac.kr

Sci. Adv. **11**, eadz3605 (2025)
DOI: 10.1126/sciadv.adz3605

The PDF file includes:

Supplementary Note S1
Figs. S1 to S32
Tables S1 and S2
Legends for movies S1 to S3
References

Other Supplementary Material for this manuscript includes the following:

Movies S1 to S3

Supplementary Note 1. Density Functional Theory (DFT) Simulation modeling workflow

The slab models exhibited dangling bonds on the vacuum-exposed surface, which were passivated using pseudo-hydrogen atoms with appropriate fractional charges to prevent the formation of surface states. To determine the vacuum level, dipole corrections were applied to compensate for the artificial dipole moment generated at the open ends (with a 20 Å vacuum space along the c-axis), arising from the periodical boundary conditions imposed in these calculations.

To identify an ideal hetero-interface, various combinations of substrate and film Miller indices were investigated (*72*). Based on criteria such as mismatch strain, supercell area, and lattice vector length, domain-matched heterostructures between the silicon carbide (SiC) and graphene with lattice mismatches below 4% were selected (fig. S4c). A similar matching procedure was used to construct interfaces between SiC/graphene and the metal layers.

To emulate the graphene-formation phenomenon during the metal-assisted graphitization (MAG) process, *ab initio* molecular dynamics (AIMD) simulations were performed in three main steps, as illustrated in fig. S5: (i) a silicon (Si)-containing metal layer was generated by substituting 10 at.% of the metal atoms with Si; (ii) the SiC/graphene/Si-dissolved metal heterostructure was relaxed through energy minimization to stabilize the initial atomic configuration of each model; and (iii) random displacements were introduced to the carbon atoms in the graphene layer to study the stabilization effects of metal catalysts arising from interface interactions between the metal and graphene layers (*30–31, 73*).

To quantify the thermodynamic driving force for interfacial sp^2 formation shown in fig. S9 (D), we calculated the grand-potential energy lowering (Ω) per interfacial carbon atom as:

$$\boldsymbol{\Omega}_{\boldsymbol{per\ C}}^{\boldsymbol{Ni+Si}} = (\langle F\rangle_{Ni+Si} - \mu_{Ni}N_{Ni} - \mu_{Si}N_{Si})/N_C,\ \boldsymbol{\Omega}_{\boldsymbol{per\ C}}^{\boldsymbol{pure\ Ni}} = (\langle F\rangle_{Ni} - \mu_{Ni}N_{Ni})/N_C,$$

where ⟨F⟩ is the DFT electronic free energy computed with a gaussian smearing, μ_{Ni} and μ_{Si} are bulk-reference chemical potentials, and N_C is the number of interfacial C atoms.

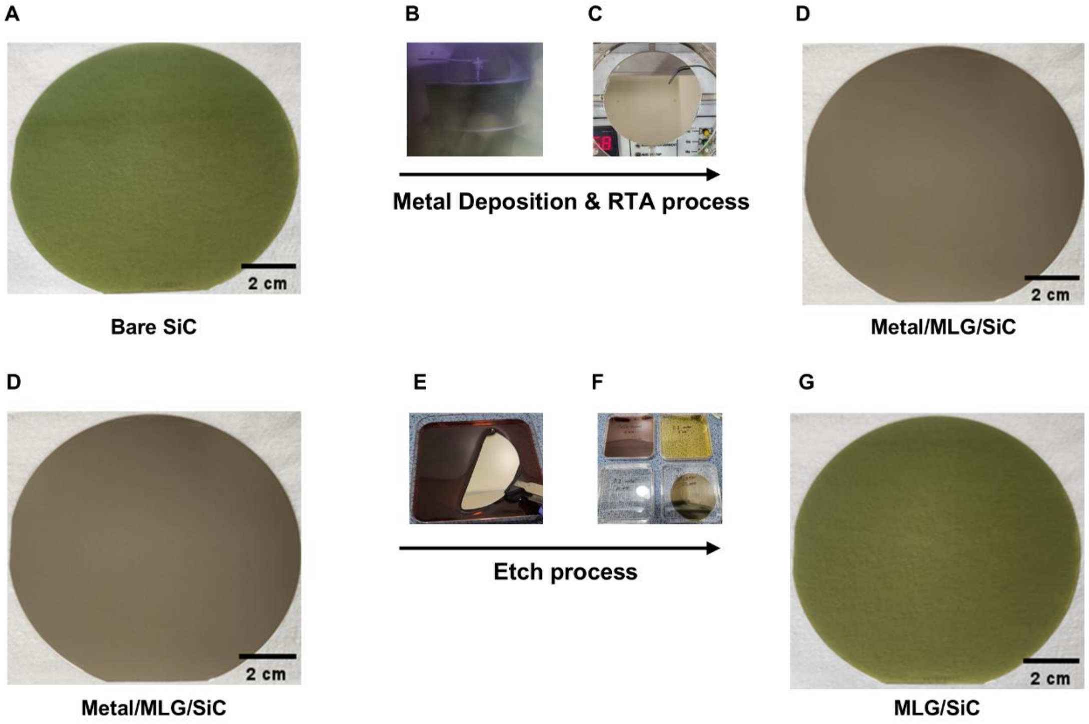

Fig. S1. Detailed metal-assisted graphitization (MAG) process. (**A**) The silicon carbide (SiC) wafer is cleaned sequentially in acetone (5 minutes), and isopropyl alcohol (IPA) for 5 minutes each using an ultrasound bath and then dried with a nitrogen gun. (**B**) Nickel (Ni) is deposited onto the SiC by sputtering at a pressure of 5×10^{-4} Torr in an argon (Ar) ambient (50 sccm) for 20 minutes. (**C**) The sample is annealed in a rapid thermal annealing (RTA) chamber under specific conditions: a heating speed of 12 °C/s, annealing for 3 minutes, and cooling by turning off the power. (**D**) Graphene forms at the Ni/SiC interface through the MAG process. (**E**) Ni is etched away using ferric chloride ($FeCl_3$) to expose the interface graphene. (**F**) Once all visible traces of Ni are removed, the sample is gently agitated in fresh $FeCl_3$, followed by rinsing in deionized water. The surface is kept wet to prevent the redeposition of Ni residues. (**G**) The final result of the MAG process demonstrates multi-layer graphene (MLG, > 2 graphene monolayers) on the entire 4-inch SiC substrate. For small-area graphene synthesis (≤ 1 cm^2), annealing at the target temperature was performed for 1 s, whereas for 4-inch wafer-scale graphitization, a prolonged annealing time of 3 min was required to achieve full graphene coverage across the wafer.

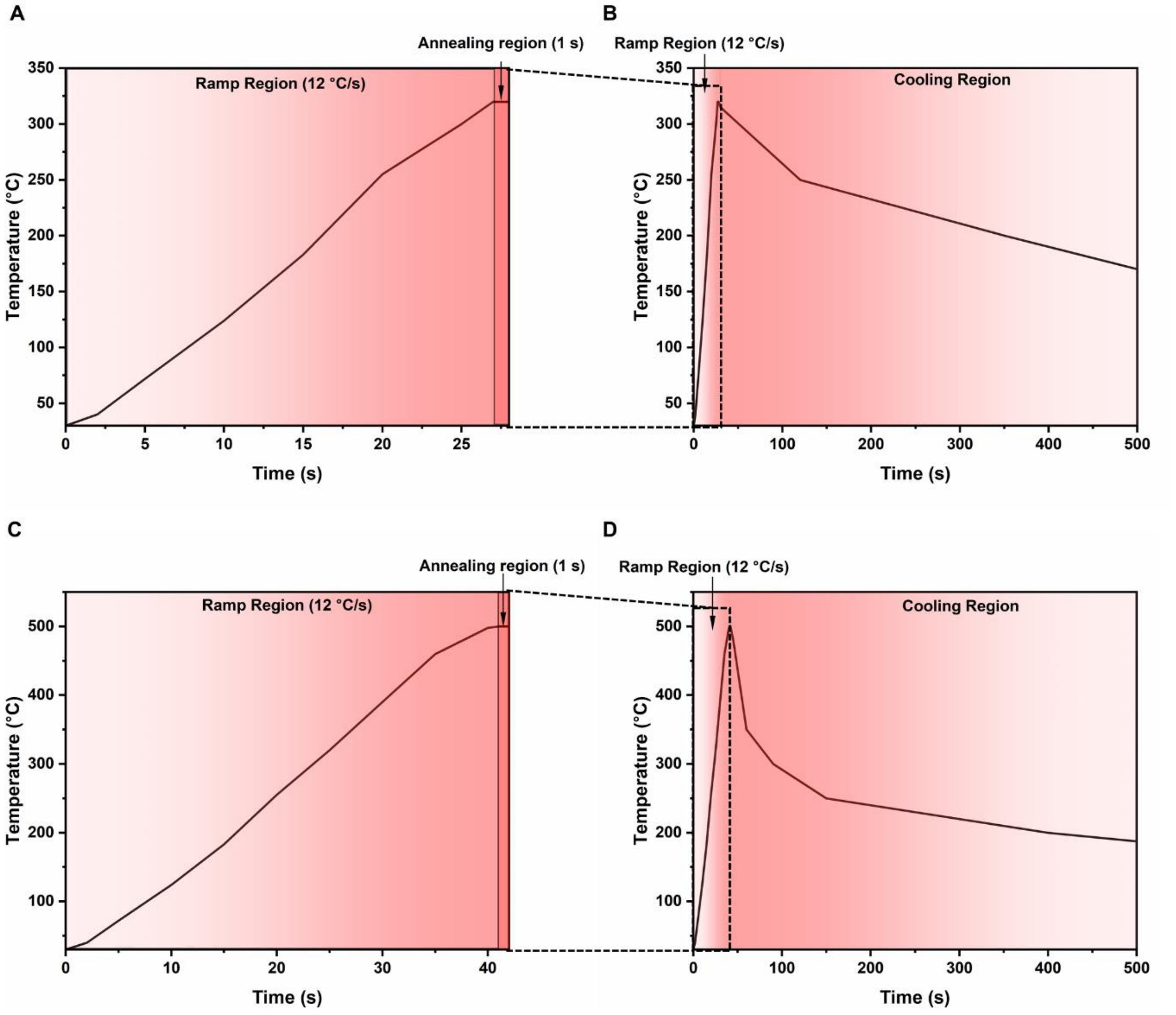

Fig. S2. Measured temperature profiles of the RTA process. Temperature-time profiles for the annealing processes used for few-layer graphene (FLG, ≤ 2 graphene monolayers) and MLG synthesis. (**A** and **B**) Ramp-up and subsequent natural cooling regions for the low-temperature process (~320 °C, ramp rate 12 °C/s, annealing time at target temperature: 1 s) used to synthesize FLG on SiC. (**C** and **D**) Ramp-up and subsequent natural cooling regions for the higher-temperature process (~500 °C, ramp rate 12 °C/s, annealing time at target temperature: 1 s) were used to synthesize MLG on SiC. The profiles were obtained by recording the thermocouple readings during representative process runs.

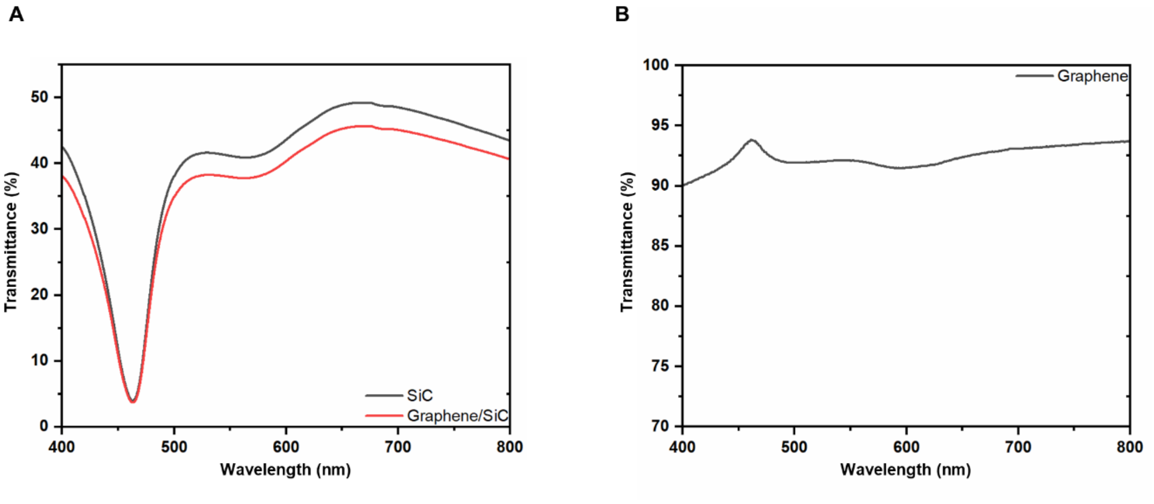

Fig. S3. Ultraviolet-visible (UV-vis) transmittance spectra in the wavelength range of 400-800 nm and criteria for FLG/MLG classification. (**A**) UV-vis transmittance spectra of SiC without graphene (black) and with graphene (red), showing reduced transmittance due to the graphene layer. (**B**) The transmittance of graphene in the visible range is measured at 92.1% at 550 nm, after subtracting the SiC background, indicating the presence of MLG on the SiC. In this study, FLG and MLG are classified based on the 550 nm transmittance: values ≥ 95.4% correspond to FLG (≤ 2 graphene monolayers), whereas values < 95.4% correspond to MLG (> 2 graphene monolayers). The absorbance of monolayer graphene corresponds to a 2.3% reduction in transmittance at 550 nm (*74*). These thresholds were cross-validated with graphene thickness measurements obtained from cross-sectional transmission electron microscope (TEM) images in the main text (Fig. 3 (C and D)).

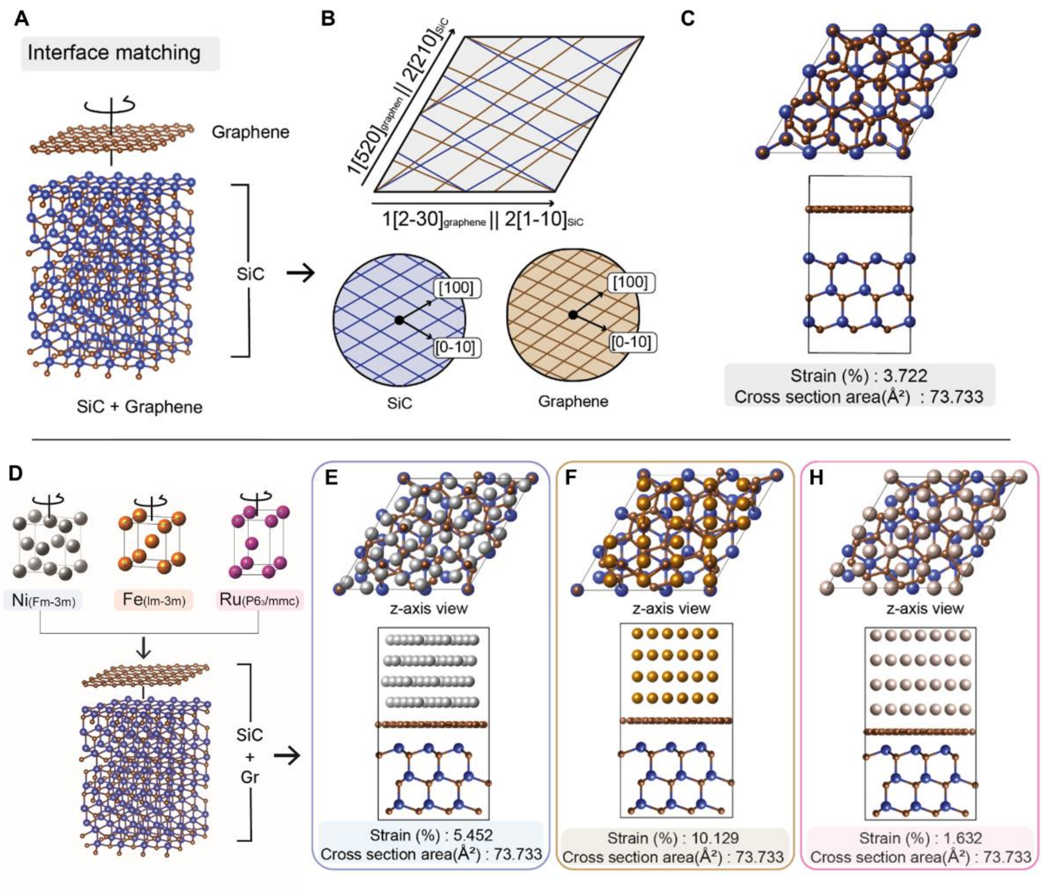

Fig. S4. Interface matching for modeling the metal/graphene/SiC structures. (**A**) Schematic illustration of interface construction for the graphene/SiC structure. (**B**) Domain-matched supercell construction for the SiC (blue) and graphene (brown) layers, with the corresponding lattice unit vectors. (**C**) Top and side views of the optimized interface configuration (Si atoms shown in blue; C atoms in dark brown). (**D**) Schematic illustration of the interface-matching process for the metal/graphene/SiC system, along with the crystal structures of Ni (Fm-3m), Iron (Fe) (Im-3m), and Ruthenium (Ru) ($P6_3$/mmc) used in the modeling. (**E** to **G**) Domain-matched heterostructures employed in the AIMD simulations: Ni (5.452% strain), Fe (10.129% strain), and Ru (1.632% strain), each with a cross-sectional area of 43.256 Å^2.

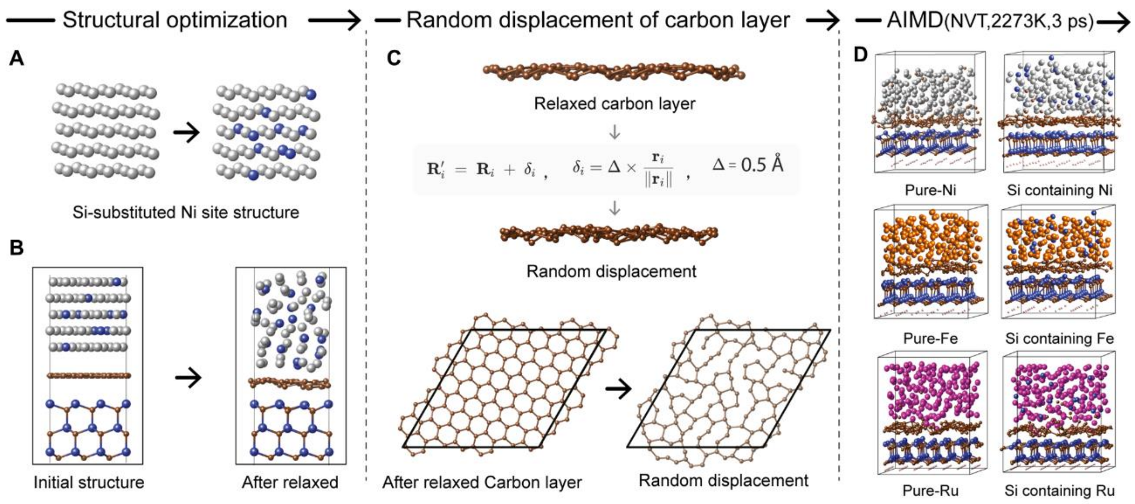

Fig. S5. Modeling workflow and *ab initio* molecular dynamics (AIMD) simulations for the metal/graphene/SiC heterostructures. (**A**) Formation of Si-containing metal layers. (**B**) Example of a relaxed configuration of the metal/graphene/SiC heterostructure model after energy minimization. (**C**) Introduction of randomly-displaced carbon atoms in the graphene layer to study the stabilization effects of metal catalysts. (**D**) Model configurations used in this study. AIMD simulations were performed for both pure and Si-containing metal layers for each metal.

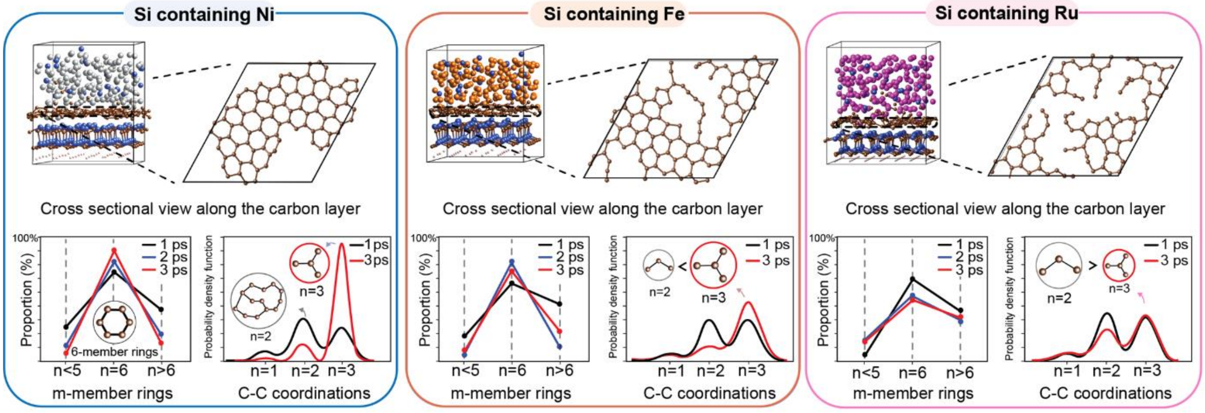

Fig. S6. AIMD simulations of Si-alloyed metal layers. Structural evolution of a carbon layer at the metal-SiC interface during AIMD simulations. The overall 3D view and top view emphasize the differences in the trajectories of carbon atoms at the interface depending on the metal elements. The computed changes in the m-membered rings centered on carbon atoms and coordination numbers clearly reveal that only Ni metal stabilizes a graphene-like structure at the interface, while this stabilization effect is not evident for Fe and Ru metals.

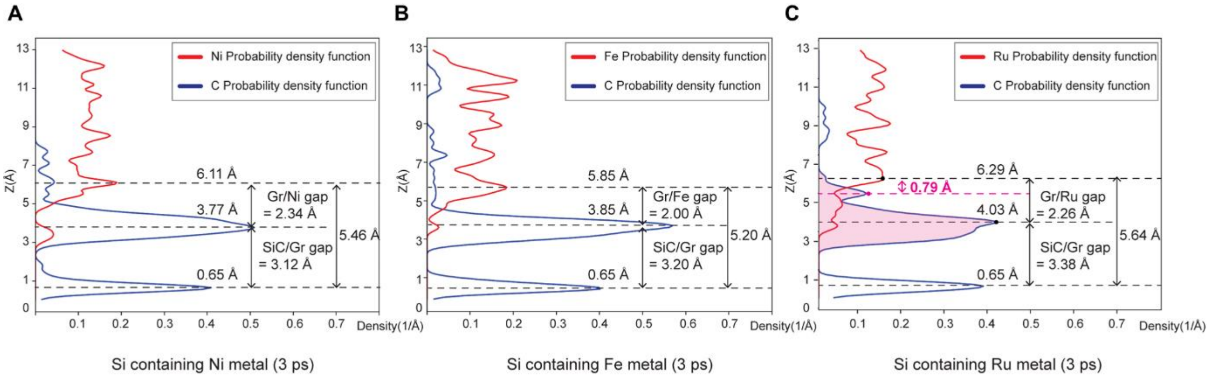

Fig. S7. Study on the van der Waals gap between the metal and carbon layers using the probability-density function. The width of the van der Waals gap observed in (**A**) the Si-dissolved Ni model, (**B**) the Si-dissolved Fe model, and (**C**) the Si-dissolved Ru model. The Ni-containing model maintained a consistent van der Waals gap between the metal and the topmost carbon layer, whereas the Fe- and Ru-containing models exhibited a collapse of the gap.

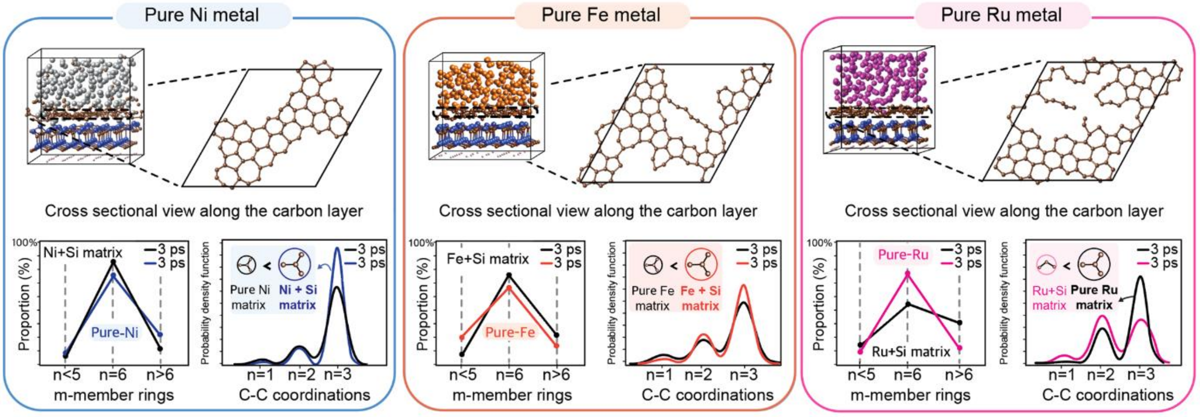

Fig. S8. AIMD simulations of metal layers without Si alloying. For pure Ni and Fe models, the number of six-fold rings and three-fold-coordinated carbon atoms was consistently lower than in their Si-dissolved counterparts, underscoring the critical role of dissolved silicon in enhancing the structural transition toward graphene-like configurations. In contrast, graphene formation in the Ru matrix occurs only when silicon is not dissolved in the Ru bulk. Among the models studied, only the Si-dissolved Ni model provided an environment where carbon atoms could readily reorganize, enabling the formation of stable, two-dimensional graphene configurations.

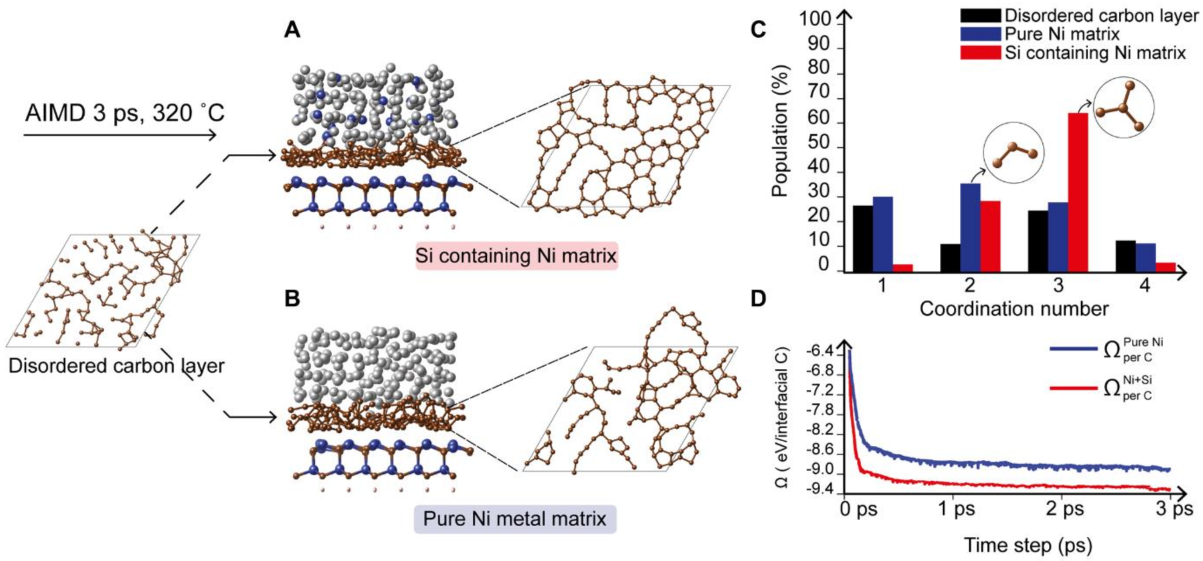

Fig. S9. Structural evolution of a disordered carbon layer at the Ni-SiC interface during AIMD simulations. AIMD simulations were performed at 593 K (~320 °C) for 3 ps to investigate the effect of Si alloying in the Ni matrix. The initial carbon source was prepared by introducing random atomic displacements (Δ=1.0 Å) to a pristine graphene layer to create a structurally disordered carbon layer, following the procedure illustrated in fig. S5c. (**A**) Snapshot of the SiC/disordered carbon/Ni-Si system, showing rapid conversion of interfacial carbon into threefold (sp^2-like) coordination networks. (**B**) Snapshot of the SiC/disordered carbon/Ni system, where the interfacial carbon remains largely disordered throughout the simulation. (**C**) Evolution of the coordination number distribution of interfacial carbon atoms, demonstrating that Si incorporation accelerates the stabilization of sp^2 bonds. (**D**) Calculated grand-potential energy lowering (Ω) per interfacial carbon atom, showing that the Ni–Si system exhibits a considerably reduced interfacial energy compared to pure Ni. Together, these results indicate that Si alloying in Ni provides a thermodynamic driving force that facilitates the crystallization of interfacial carbon into ordered graphene layers at low temperatures (~320 °C). Ω was defined as described in Supplementary Note 1.

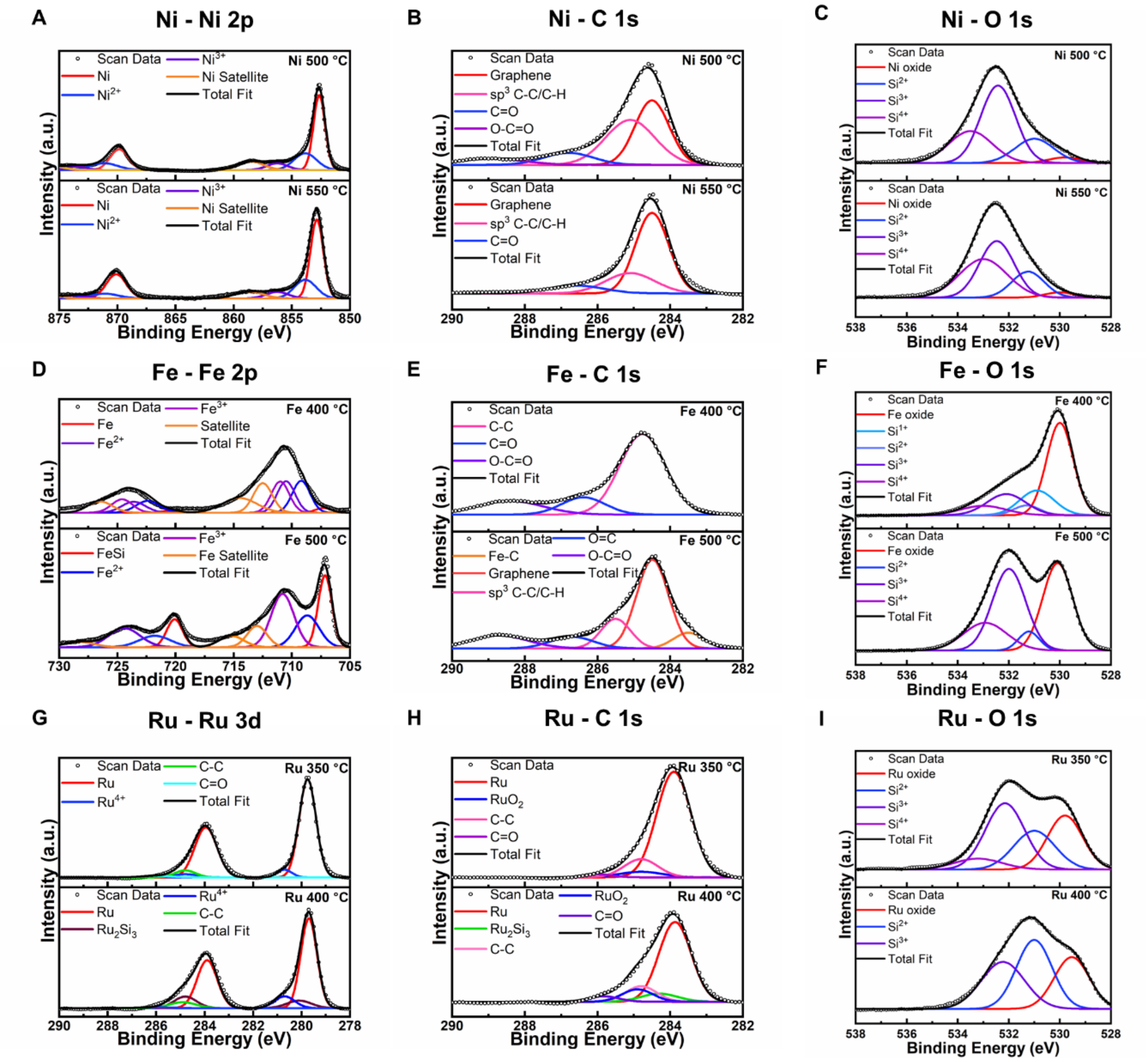

Fig. S10. X-ray photoelectron spectra (XPS) of metal/SiC after annealing at various temperatures. (**A** to **C**) Ni/SiC: Ni 2p, C (carbon) 1s, and oxide (O) 1s spectra after annealing at 500 °C and 550 °C. (**D** to **F**) Fe/SiC: Fe 2p, C 1s, and O 1s spectra after annealing at 400 °C and 500 °C. (**G** to **I**) Ru/SiC: Ru 3d, C 1s, and O 1s spectra after annealing at 350 °C and 400 °C. All spectra were calibrated using the C-C bond (284.8 eV) or C=C bond (284.6 eV) and cross-checked with Raman spectra (see fig. S11) and X-ray diffraction (XRD) (see fig. S12) results. Additional calibration was performed using the O 1s peak, which reflects oxidation induced during and after annealing. The thickness of the deposited metal layer exceeded the detection limit depth of XPS (typically around 10 nm), indicating that the detected Si and C atoms originated from the SiC.

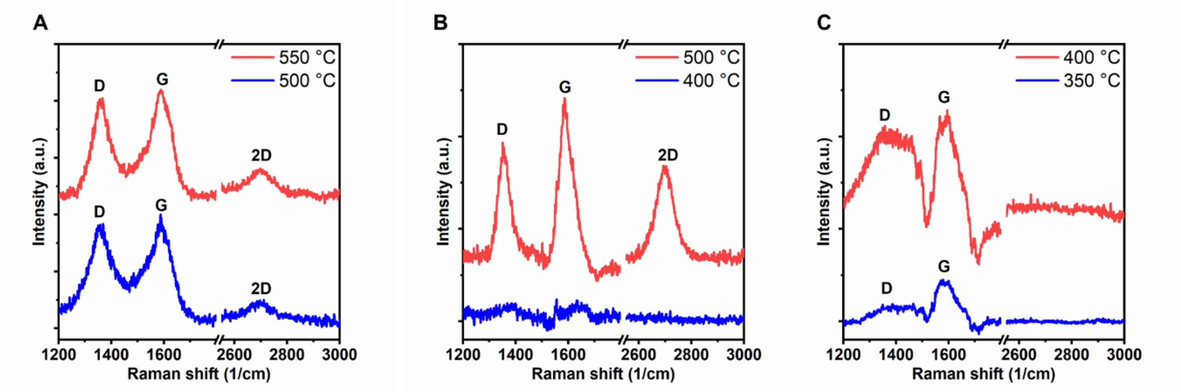

Fig. S11. Raman spectra after annealing at corresponding temperatures. (**A**) Comparison of the Ni/SiC Raman spectra at different annealing temperatures. (**B**) Comparison of the Fe/SiC Raman spectra at different annealing temperatures. (**C**) Comparison of the Ru/SiC Raman spectra at different annealing temperatures. At 500 °C for Ni, 500 °C for Fe, and 400 °C for Ru, liberated carbon diffused outward and formed graphitic carbon layers on the metal surface, consistent with XPS results. For Ni, Raman and XRD measurements (fig. S12) confirm the presence of graphene on the metal surface without any silicide formation under 500 °C. However, for Fe and Ru, the Raman and XRD data indicate that graphitic carbon formation on the surface occurred only after silicide formation. For Fe, the pronounced 2D (~2700 cm^{-1}) peak in the Raman spectra suggests the formation of relatively crystalline graphene, whereas Ru shows only broad D (~1350 cm^{-1}) and G (~1580 cm^{-1}) peaks, consistent with an amorphous-like graphene layer. All samples were subjected to a prolonged 3-minute annealing process at the corresponding temperatures.

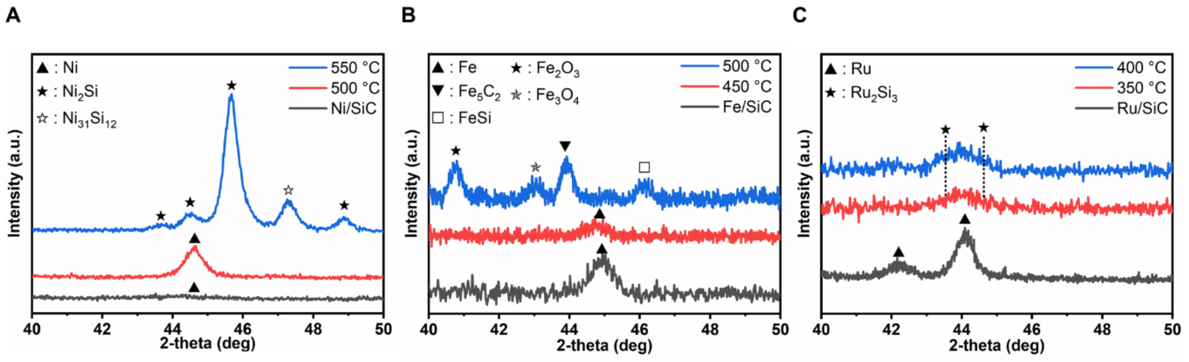

Fig. S12. XRD patterns after annealing at corresponding temperatures. The following XRD patterns were obtained from a 50 nm thick metal on SiC after a prolonged 3-minute anneal at various temperatures. (**A**) Comparison of the Ni/SiC XRD patterns at various annealing temperatures. The data clearly shows that no Ni-silicide phases are formed at 500 °C (red curve). The characteristic peaks for Ni_2Si and $Ni_{31}Si_{12}$ appear only after annealing at the higher temperature of 550 °C (blue curve). The reference patterns correspond to JCPDS no. 71-4655 for Ni, JCPDS no. 79-3559 for Ni_2Si, and JCPDS no. 17-0222 for $Ni_{31}Si_{12}$. (**B**) Comparison of the Fe/SiC XRD patterns at various annealing temperatures. The FeSi phase is observed only after annealing at 500 °C (blue curve). The reference patterns correspond to JCPDS no. 76-6588 for Fe, JCPDS no. 79-3559 for FeSi, JCPDS no. 89-8103 for Fe_2O_3, JCPDS no. 71-6336 for Fe_3O_4, and JCPDS no. 89-8968 for Fe_5C_2. (**C**) Comparison of the Ru/SiC XRD patterns at various annealing temperatures. The Ru_2Si_3 phase is already present at the lowest annealing temperature of 350 °C (red curve) and persists at 400 °C (blue curve). The reference patterns correspond to JCPDS no. 73-7011 for Ru, and JCPDS no. 88-0895 for Ru_2Si_3.

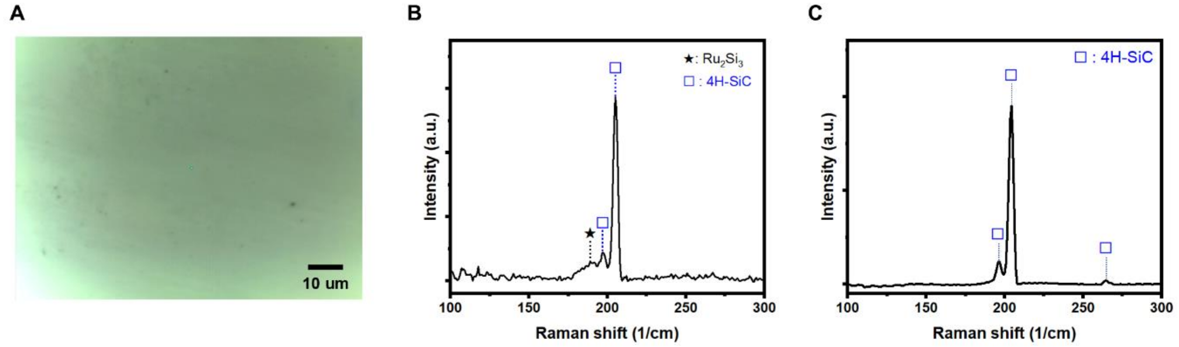

Fig. S13. Optical image and Raman spectra of the Ru/SiC annealed at 350 °C. (**A** and **B**) Raman spectra with optical image reveal the presence of Ru_2Si_3 peak at 203 cm^{-1}, consistent with XPS and XRD results. (**C**) Raman spectra of bare SiC in the range of 50 cm^{-1} to 500 cm^{-1}, indicate the absence of the 203 cm^{-1} peak.

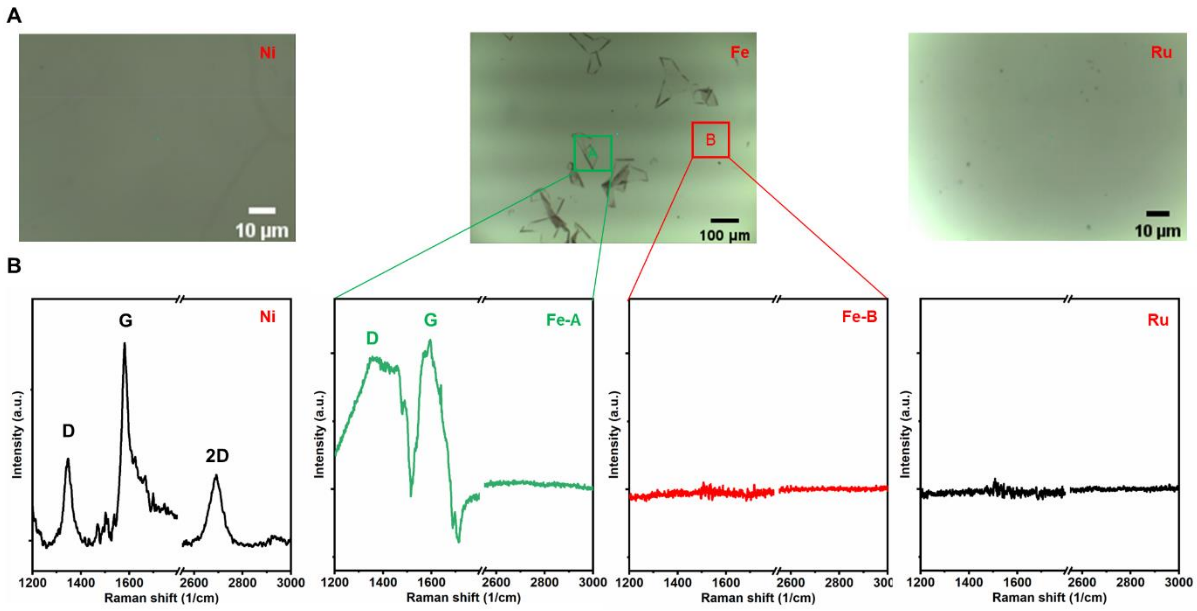

Fig. S14. Comparative analysis of different metal catalysts for the MAG process after metal removal. (**A**) Optical images of SiC surfaces after annealing with Ni at 500 °C, Fe at 400 °C, and Ru at 350 °C, followed by complete removal of the residual metal using the appropriate etchant. The faint lines visible in the optical image for the Ni system are attributed to wrinkles formed during the cooling process. (**B**) Corresponding Raman spectra from each sample after metal removal. The Ni system shows all characteristic graphene peaks (D, G, and 2D). In contrast, the Fe system exhibits a disordered, spatially non-uniform graphitic layer without a discernible 2D peak. The Ru system shows no detectable graphene-related Raman peaks after etching. These results collectively demonstrate that, under these conditions, Ni is the only effective catalyst for synthesizing a stable graphene layer at the metal/SiC interface.

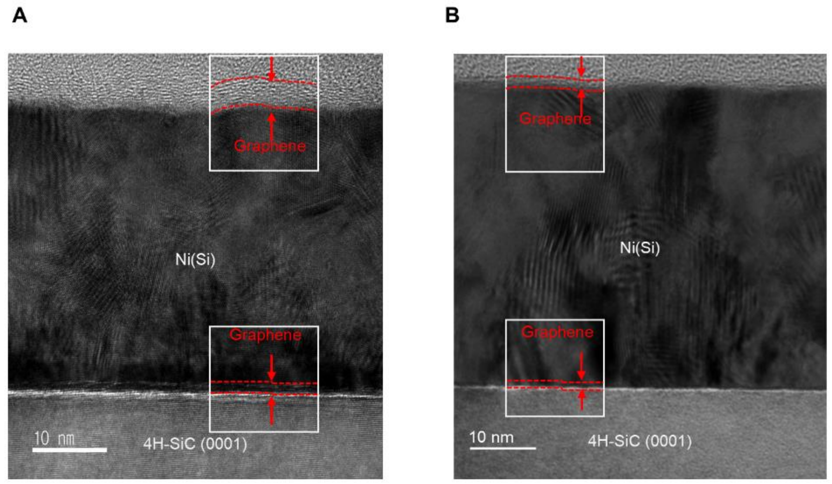

Fig. S15. Cross-sectional TEM analysis of the Ni/SiC system at different annealing temperatures. (**A**) At 500 °C, a thicker graphene layer is observed at both the Ni(Si) surface and the Ni(Si)/SiC interface. (**B**) At 320 °C, a thinner graphene layer is visible at both locations. Direct comparison of the two conditions demonstrates that graphene thickness increases with annealing temperature, confirming that the MAG process is primarily temperature-driven. The interfacial graphene thicknesses observed here are further quantified in fig. S16 by UV-vis transmittance spectroscopy, using the FLG/MLG classification criteria described in fig. S3.

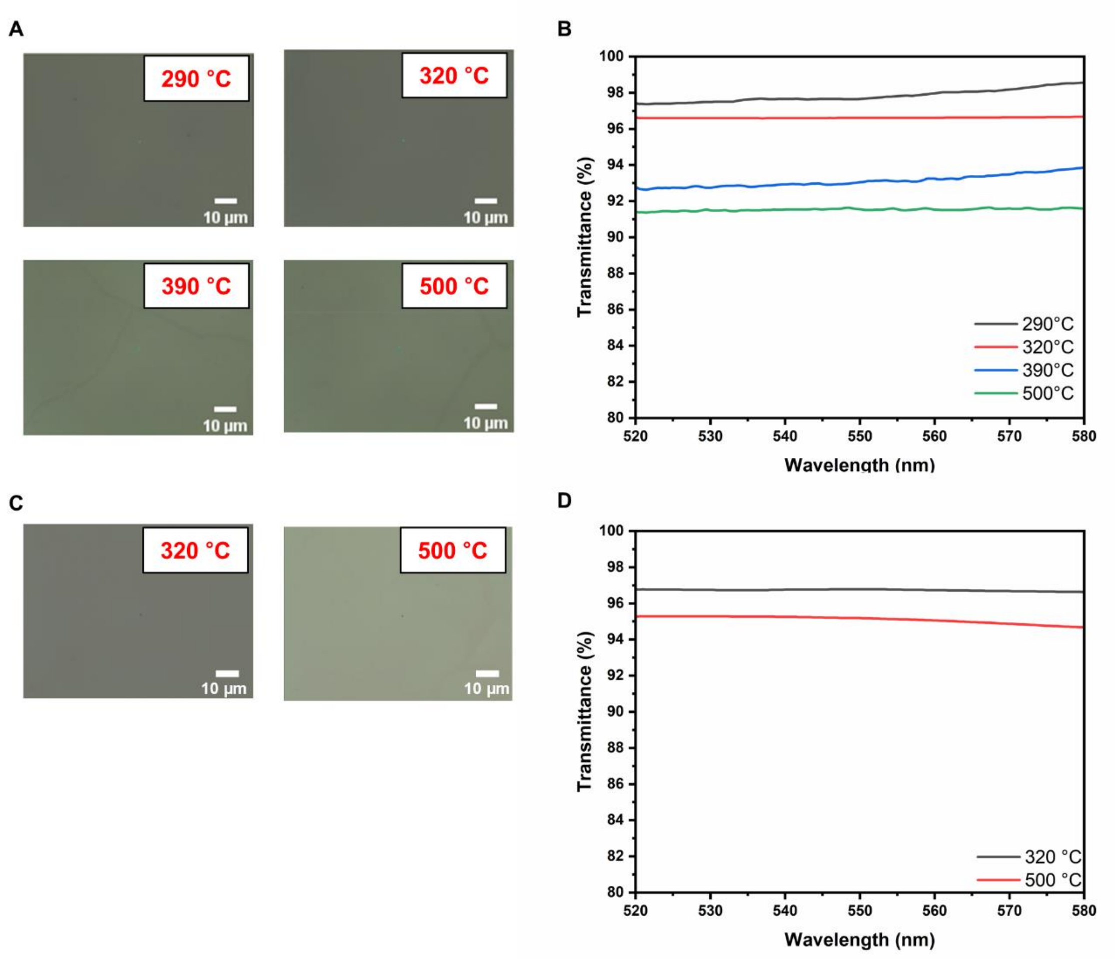

Fig. S16. Optical images and UV-vis transmittance spectra of graphene synthesized via the MAG process using Ni films deposited by sputtering and electron-beam evaporation. (**A** and **B**) Optical images and UV-vis transmittance spectra of graphene synthesized using sputtered Ni (50 nm) after annealing at the indicated temperatures and Ni removal. At 320 °C, the measured transmittance corresponds to FLG, while at 500 °C, it corresponds to MLG, based on the classification criteria in fig. S3. These results are consistent with TEM observations in fig. S15, which show thicker graphene at higher annealing temperatures. Wrinkles in the optical images are attributed to graphene wrinkles formed during the cooling stage. (**C** and **D**) Optical images and UV-vis transmittance spectra of graphene synthesized using e-beam evaporated Ni (50 nm) annealed at 320 °C and 500 °C. The 320 °C sample corresponds to FLG, whereas the 500 °C sample corresponds to MLG. Higher transmittance compared to sputtered Ni indicates reduced graphene thickness (~1 monolayer thinner at 320 °C and ~2 ML thinner at 500 °C), possibly due to the lower residual stress or grain size differences of the e-beam evaporated Ni. All spectra are shown after SiC background subtraction.

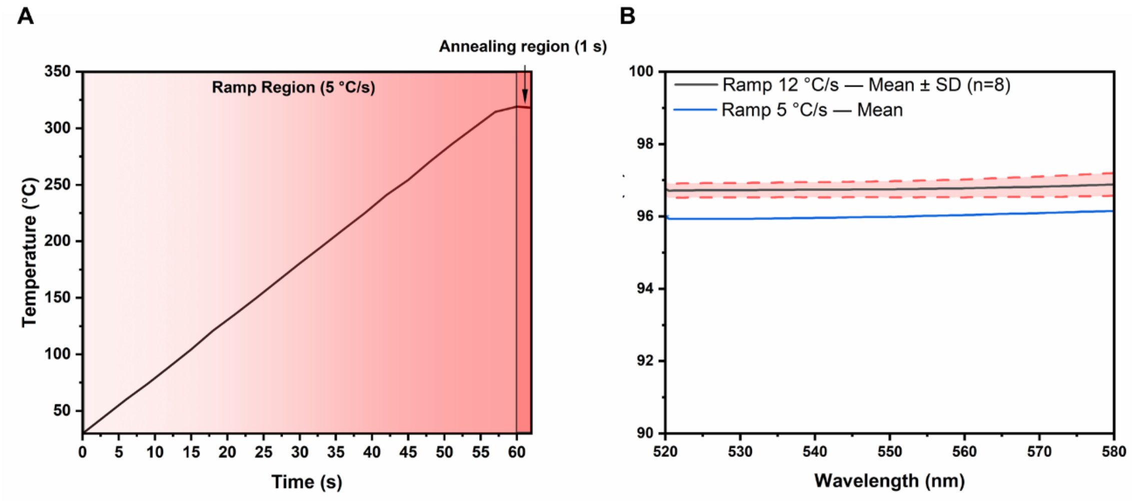

Fig. S17. RTA temperature profile and UV-vis transmittance spectra for different ramp rates. (**A**) Temperature–time profile for RTA at 320 °C with a ramp rate of 5 °C/s, including a 1 s annealing hold at the target temperature. (**B**) UV-vis transmittance spectra of Ni (50 nm) films after RTA at 320 °C with ramp rates of 12 °C/s (mean ± standard deviation (SD), n = 8) and 5 °C/s (mean). A decrease in ramp rate from 12 °C/s to 5 °C/s was accompanied by a reduction in transmittance at 550 nm from 96.75% to 96.00%, suggesting a slight but not substantial increase in graphene thickness. In both cases, the measured transmittance confirms that the films remain within the FLG range. For 12 °C/s condition, the SD of 0.23% indicates high reproducibility; however, the lower transmittance of the 5 °C/s sample lies outside this variation range. All spectra are presented after subtracting the SiC substrate background. The red dashed lines in panel b represent the standard deviation (mean ± SD) of the UV–vis transmittance spectra for the 12 °C/s condition.

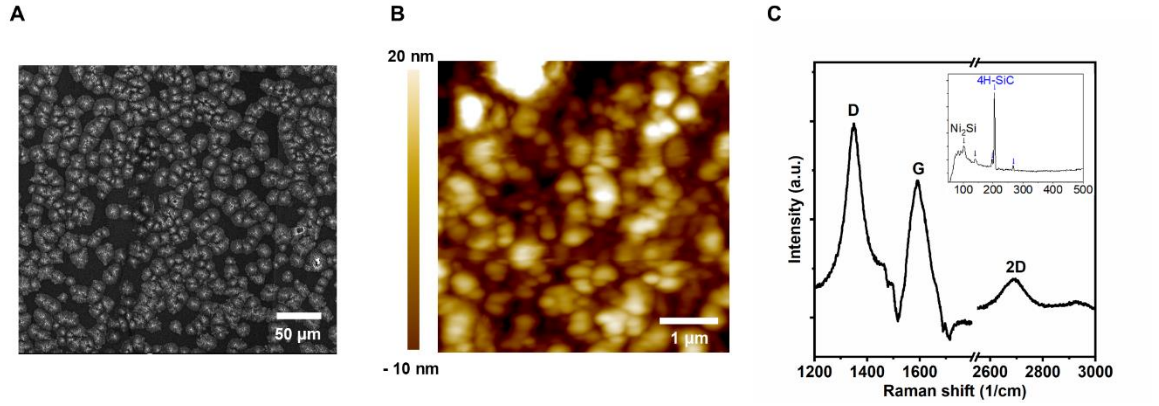

Fig. S18. Scanning Electron Microscopy (SEM), Atomic Force Microscopy (AFM) images, and Raman spectra of the SiC surface after annealing above the silicide formation temperature. (**A** and **B**) SEM and AFM images of a sample annealed at 550 °C followed by Ni removal using $FeCl_3$ solution. The surface exhibits inhomogeneous reactions, with the formation of a non-uniform, clustered film on the SiC surface. The AFM analysis shows a root-mean-square roughness (R_q) of 10.54 nm, reflecting the presence of these clustered features. (**C**) Raman spectra from the clustered regions exhibit the characteristic D, G, and 2D peaks of graphene, together with a background signal from nickel silicide (Ni_2Si), indicating that the clusters consist of graphene in the presence of an underlying Ni_2Si phase. This indicates that, above the silicide formation temperature, undesirable interfacial reactions occur between Ni and SiC, degrading the uniformity of the graphene layer. These findings highlight the necessity of performing the MAG process at annealing temperatures below the silicide formation threshold to avoid SiC surface and graphene degradation.

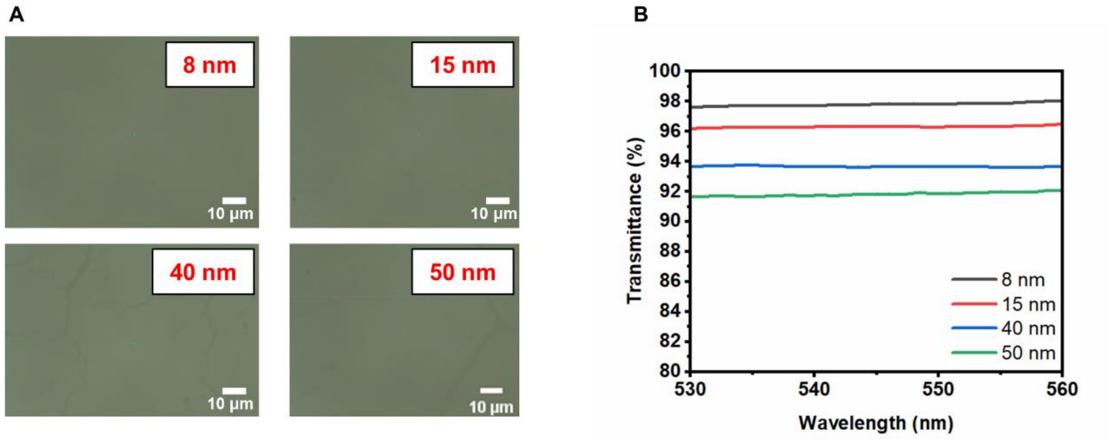

Fig. S19. Control of graphene thickness by varying the initial Ni catalyst thickness. (**A**) Optical images of samples prepared with different initial Ni thicknesses (8, 15, 40, and 50 nm) after annealing at 500 °C and subsequent Ni removal using $FeCl_3$ solution. The faint lines visible in the images are wrinkles, which form to relieve compressive strain during the cooling process. (**B**) Corresponding UV-vis spectra show that transmittance at 550 nm increases as the Ni layer becomes thinner, indicating a reduction in graphene thickness. According to the classification criteria in fig. S3, Ni thicknesses of 8 and 15 nm yield FLG, whereas 40 and 50 nm produce MLG. This confirms that the final graphene thickness is directly dependent on the initial catalyst thickness, consistent with the catalytic role of Ni in promoting Si-C bond dissociation. All spectra are presented after subtracting the SiC substrate background.

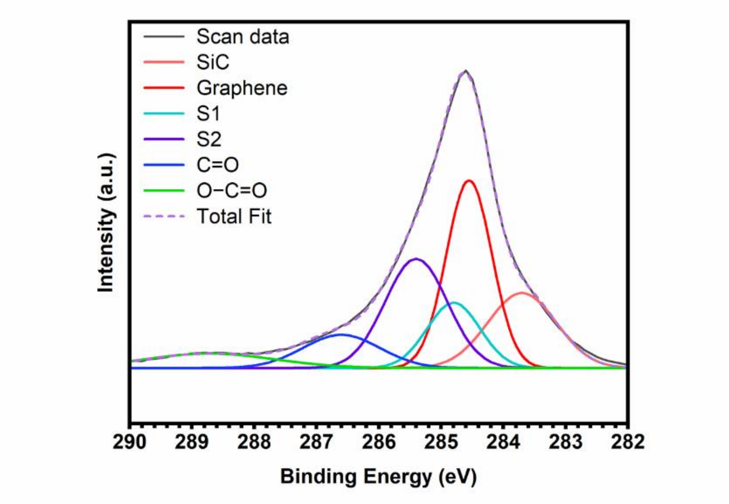

Fig. S20. C 1s XPS spectrum of the FLG/SiC sample. The spectrum was obtained from a sample prepared with an initial 50 nm Ni layer, annealed at 320 °C with a ramp rate of 12 °C/s, held for 1 s at the target temperature, and subsequently etched. The deconvoluted C 1s spectrum shows distinct peaks corresponding to the SiC substrate (283.7 eV), sp^2 graphene (284.5 eV), and the characteristic graphene buffer layer (GBL) components, S1 (284.8 eV) and S2 (285.4 eV), along with minor C=O and O–C=O contributions. The presence of these components suggests that the MAG-treated FLG sample possesses the FLG/GBL/SiC structure.

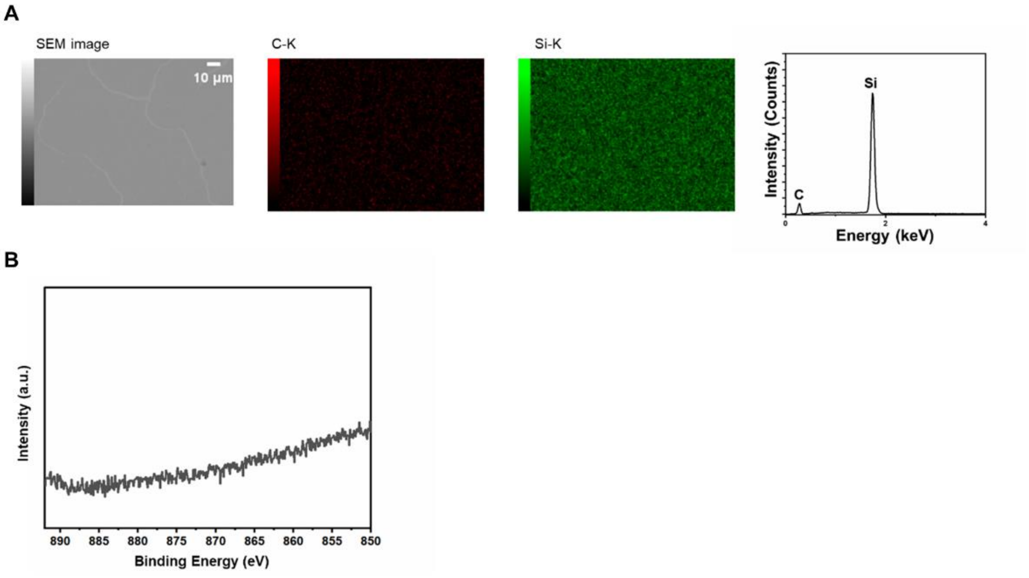

Fig. S21. Energy Dispersive Spectroscopy (EDS) and XPS analyses confirming complete removal of the Ni catalyst. (**A**) SEM image of the graphene/SiC surface after Ni removal by $FeCl_3$ etching, along with the corresponding C and Si EDS elemental maps and EDS spectrum. The fine, parallel lines visible in the SEM image are graphene wrinkles formed during the cooling stage. (**B**) High-resolution Ni 2p XPS spectrum from the same surface shows no detectable Ni signal. Both EDS and XPS analyses confirm the complete removal of the Ni catalyst.

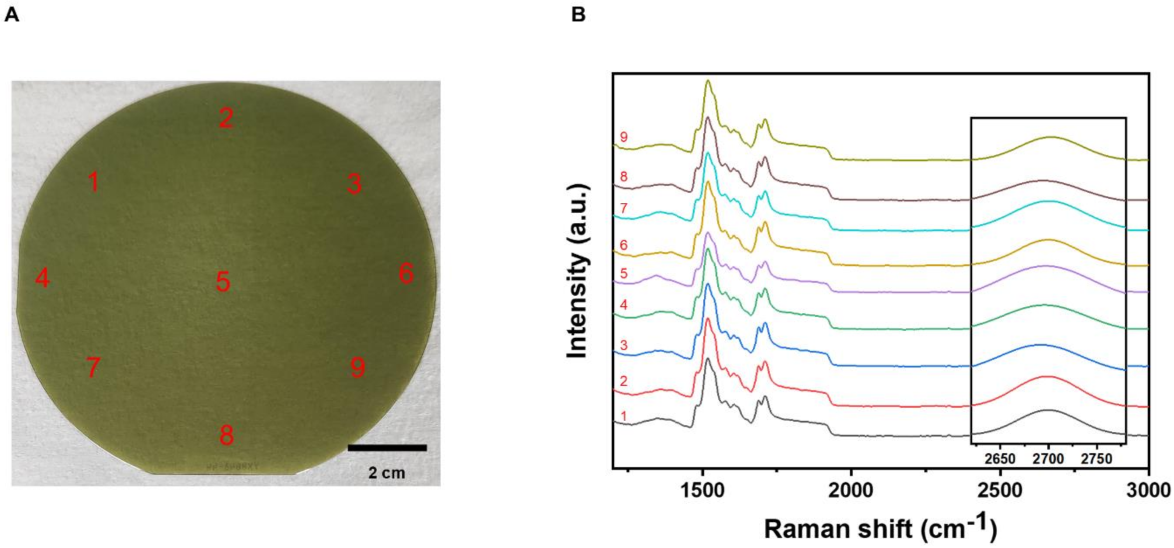

Fig. S22. Raman spectra of MAG-synthesized graphene on 4-inch wafer. (**A**) Photograph of a fully graphitized 4-inch SiC wafer obtained using the conditions described in fig. S1. (**B**) Raman spectra collected from nine representative positions (marked in panel A) exhibit the characteristic D, G, and 2D peaks of graphene; the overall presence of graphene features across the wafer demonstrates large-area coverage.

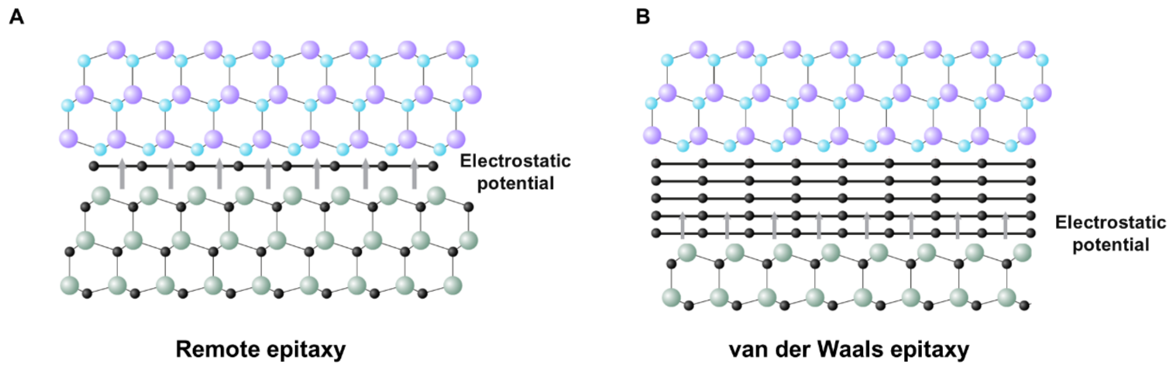

Fig. S23. Schematic illustration of 2D-assisted epitaxy mechanisms. 2D-assisted epitaxy encompasses techniques like remote epitaxy and van der Waals epitaxy (vdWE), which enable high-quality material growth on 2D materials. However, these techniques differ substantially in their requirements for preparing the graphitized sample. Remote epitaxy requires FLG that is robust enough to withstand the harsh growth environment while allowing the lattice information of the underlying substrate to guide the growth of the crystalline film. In contrast, vdWE does not rely on the lattice information of the substrate. Instead, it demands MLG to completely screen the substrate's electrostatic potential, which enhances the quality of the grown membrane by eliminating substrate interference (*48*).

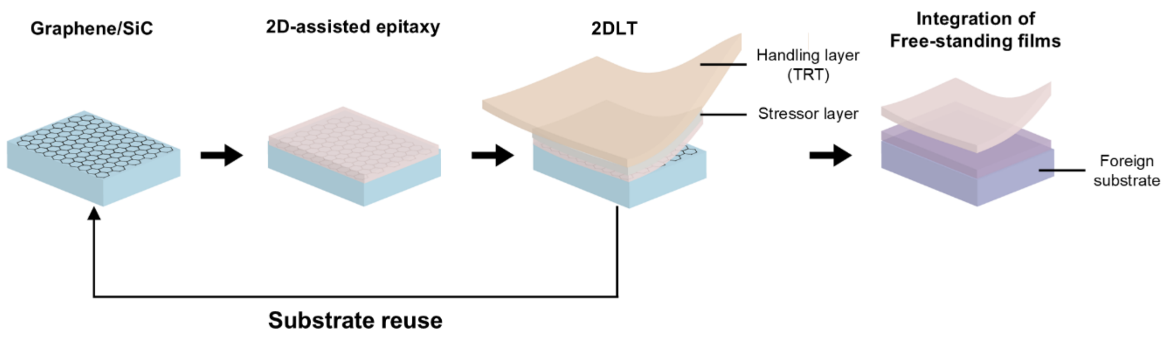

Fig. S24. Schematic illustration of 2D-assisted epitaxy process. The process begins with the graphitization of the SiC to synthesize graphene. This graphitized template is then prepared for 2D-assisted epitaxy, where a wide-bandgap material is grown using a growth chamber, such as metal-organic chemical vapor deposition (MOCVD) or molecular beam epitaxy (MBE), under optimized conditions. After the growth, a stressor layer, typically Ni, is deposited via a sputtering or electron-beam evaporator. The thickness and deposition conditions of the Ni layer must be carefully controlled to enable precise exfoliation of the membrane at the van der Waals gap between the membrane and the graphitized SiC interface. Additionally, an adhesion layer may be employed to prevent peeling of the stressor layer. A thermal release tape (TRT) is then applied as a handling layer, and exfoliation is carried out by smoothly lifting the handling layer. This step corresponds to the 2D material-assisted layer transfer (2DLT) process, in which the presence of graphene enables damage-free delamination at the van der Waals interface, allowing the overgrown membrane to be reused or integrated on a foreign substrate. Finally, the TRT is removed by heating the membrane-attached-foreign-sample above 110 °C.

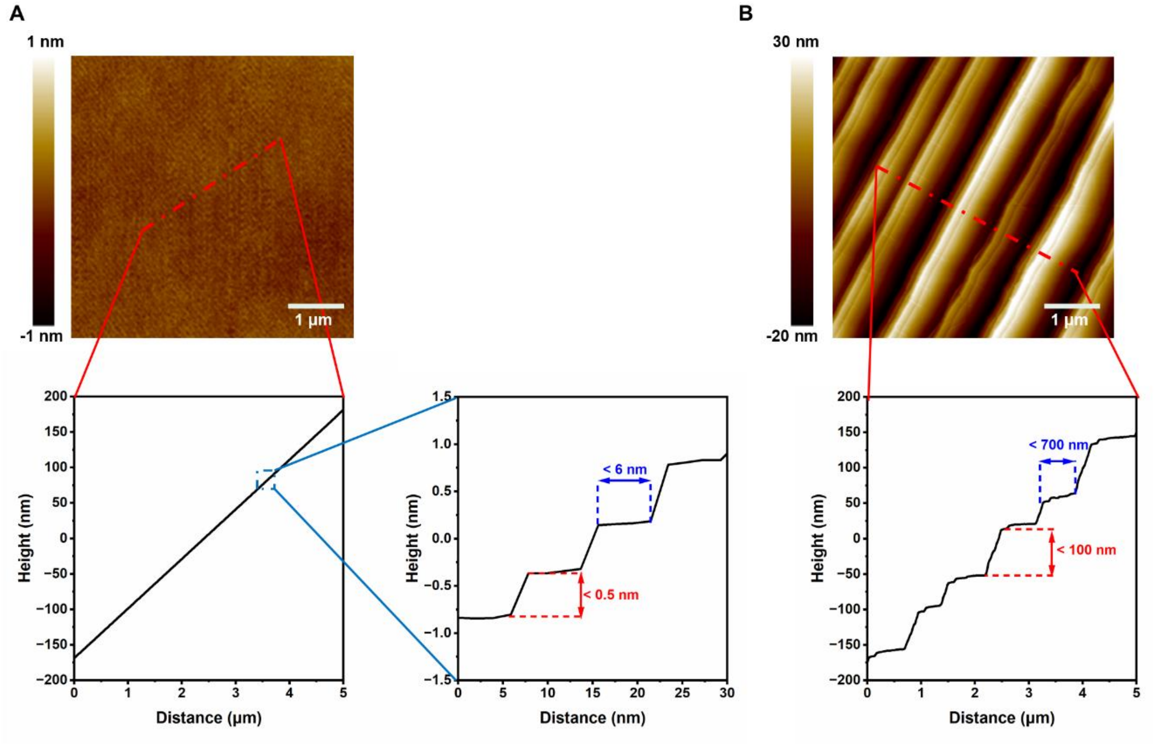

Fig. S25. Comparison of surface morphology for MAG-treated versus high-temperature graphitized SiC. (**A**) AFM images and corresponding line profiles (along the red line) reveal terrace widths below 6 nm and step heights of approximately 0.5 nm. (**B**) In contrast, the surface of a conventionally high-temperature graphitized SiC substrate exhibits pronounced step-bunching, resulting in micron-scale terraces and large step heights that are detrimental to the quality of subsequent epitaxial growth. This comparison highlights a key advantage of the low-temperature MAG process.

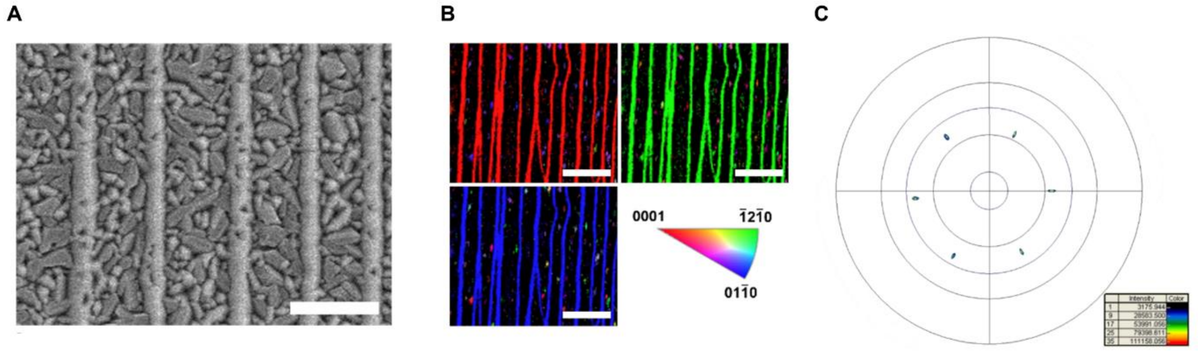

Fig. S26. Structural characterization of aluminum nitride (AlN) films grown on high-temperature graphitized SiC. (**A**) Plan-view SEM image showing rough, non-uniform AlN morphology following the step-bunched surface of graphitized SiC. The AlN grains appear misoriented and lack long-range continuity. (**B**) Electron Backscatter Diffraction (EBSD) orientation maps along different crystallographic axes, indicating the absence of uniform orientation and revealing randomly oriented grains. (**C**) XRD pole figure of the AlN {10-15} reflection, showing broad and diffuse intensity distributions rather than sharp sixfold symmetry, confirming the polycrystalline nature of the film. This behavior is in stark contrast to the highly oriented AlN films grown on MAG-treated FLG and MLG substrates, as shown in fig. S27. Scale bars, (A) 500 nm; (B) 2 μm.

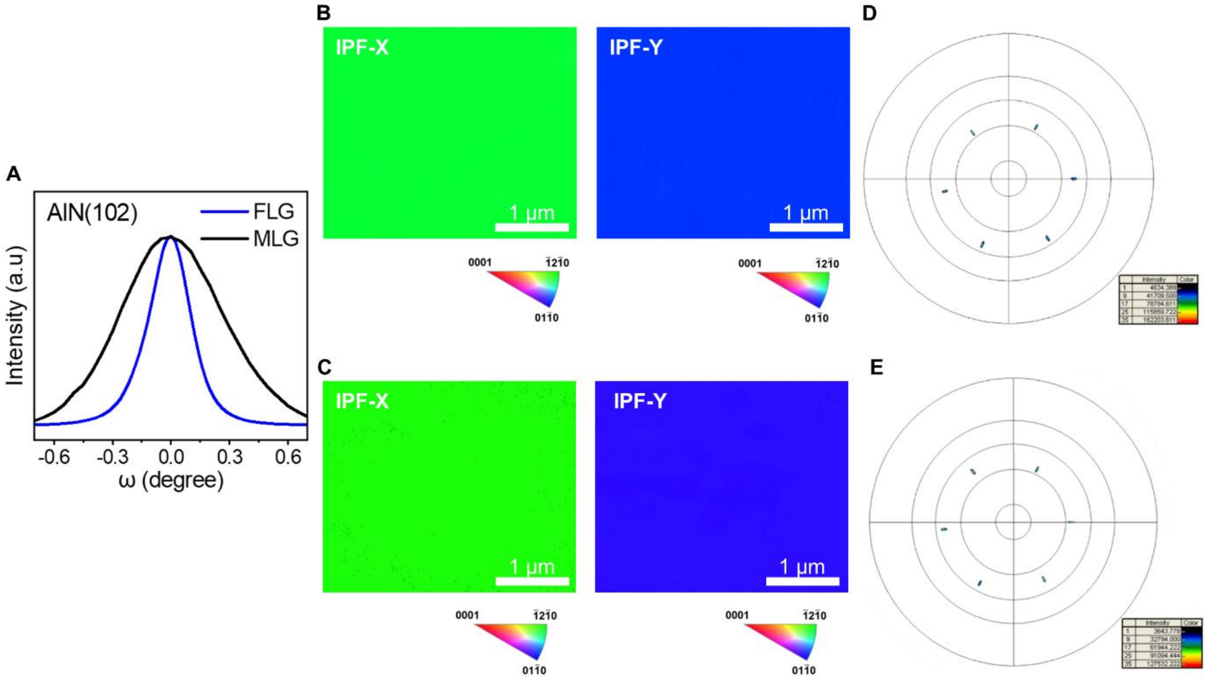

Fig. S27. Crystalline quality comparison of AlN films grown on MAG-treated FLG/SiC and MLG/SiC templates. (**A**) XRD rocking curves of the AlN (102) reflection for FLG and MLG samples, showing narrower full width at half maximum (FWHM) for FLG, indicative of higher crystalline quality. (**B** and **C**) EBSD maps of AlN grown on FLG (B) and MLG (C), confirming uniform c-axis orientation across both templates**.** (**D** and **E**) Pole figures of the asymmetric AlN {10–15} reflection for AlN on FLG (D) and MLG (E), both displaying clear sixfold symmetry characteristic of wurtzite AlN. These results, when compared with the polycrystalline growth observed on high-temperature graphitized SiC (fig. S26), demonstrate that AlN grown on both FLG and MLG exhibits strong epitaxial registry, with improved crystalline quality on FLG due to remote epitaxial interaction.

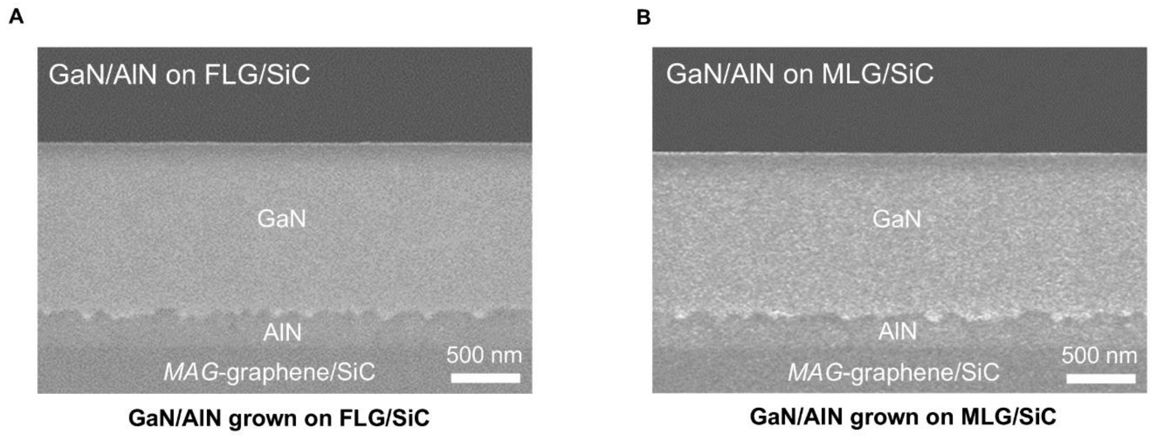

Fig. S28. Gallium nitride (GaN)/AlN heterostructure film grown on MAG-treated FLG/SiC and MLG/SiC. (**A**) Cross-sectional SEM image of GaN/AlN grown on FLG/SiC. (**B**) Cross-sectional SEM image of GaN/AlN grown on MLG/SiC. In both cases, the AlN layers consist of laterally connected grains with exposed crystalline facets rather than completely isolated islands. Both GaN/AlN heterostructures were epitaxially grown under identical MOCVD conditions.

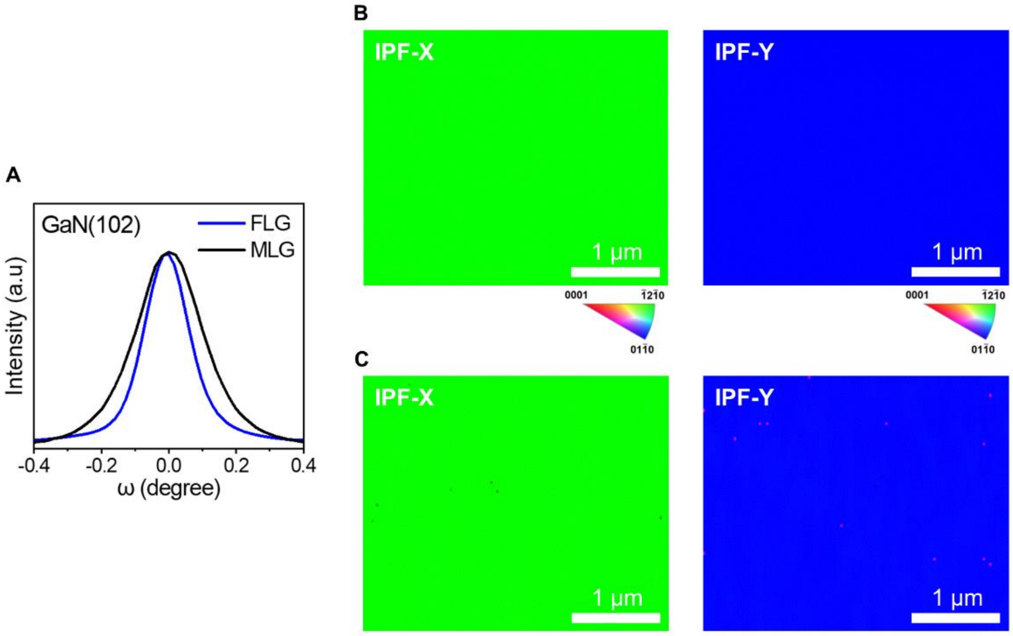

Fig. S29. HR-XRD rocking curve and In-plane EBSD of GaN/AlN grown on MAG-treated FLG/SiC and MLG/SiC. (**A**) In-plane HR-XRD rocking curve of the (102) plane, showing a FWHM of 509 arcsec for GaN on AlN/FLG/SiC and 760 arcsec for GaN on AlN/MLG/SiC, comparable to the (002) plane, which exhibits FWHM values of 270 arcsec and 444 arcsec, respectively. (**B**) In-plane EBSD maps of GaN grown on AlN/FLG/SiC. (**C**) In-plane EBSD maps of GaN grown on AlN/MLG/SiC. The EBSD patterns indicate the single-crystalline nature of the GaN film on both AlN/FLG/SiC and AlN/MLG/SiC templates.

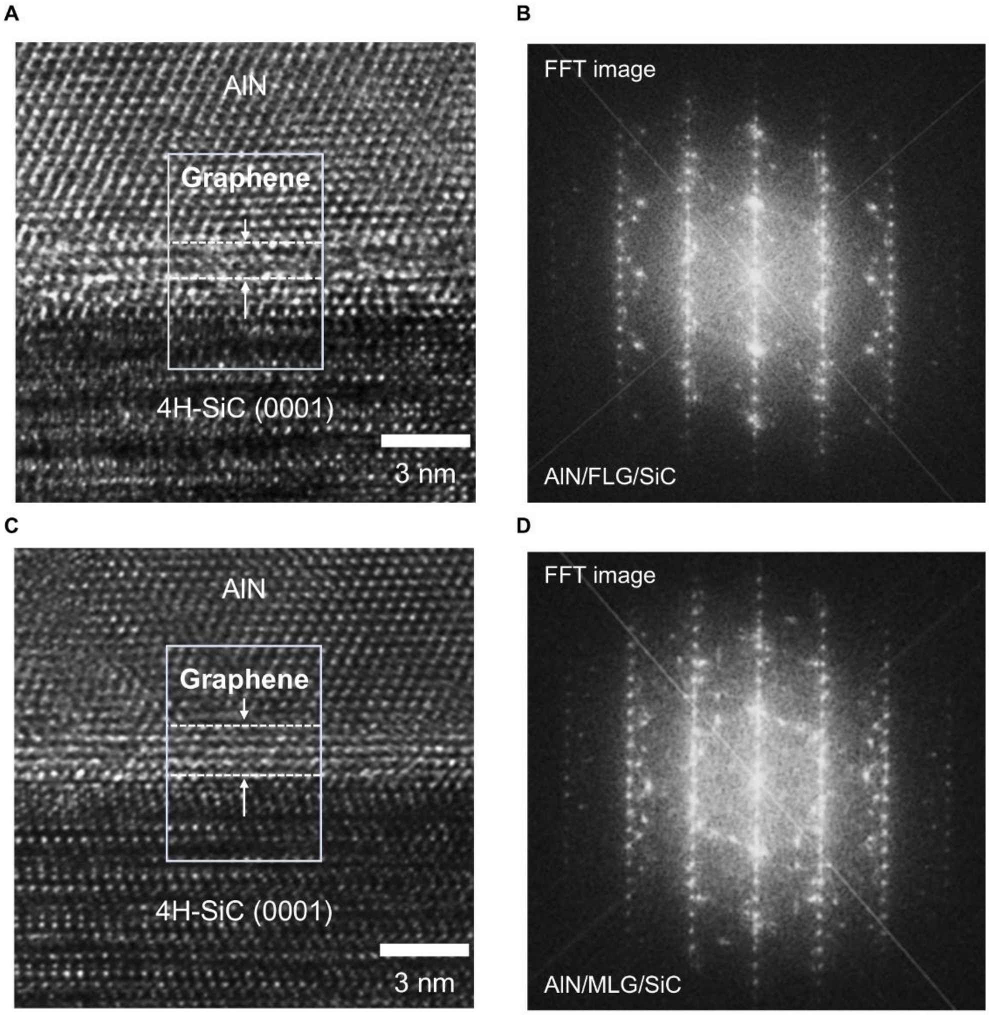

Fig. S30. Cross-sectional TEM analysis of AlN grown on MAG-treated FLG and MLG templates. (**A**) High-resolution TEM image of AlN grown on FLG/SiC substrate, where the graphene layer remains after AlN growth at the AlN/SiC interface. (**B**) Fast Fourier transform (FFT) pattern of the boxed region in panel (B), supporting remote epitaxial alignment of AlN on FLG/SiC. (**C**) High-resolution TEM image of AlN on MLG/SiC, showing that the thicker graphene layer is preserved at the interface. (**D**) FFT pattern of the boxed region in panel (C), consistent with vdWE and indicating reduced epitaxial alignment compared to the FLG case.

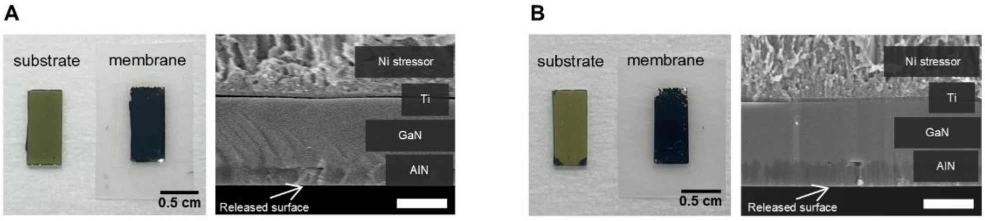

Fig. S31. Exfoliation results of GaN/AlN membranes grown on FLG/SiC and MLG/SiC templates. (**A** and **B**) Camera and SEM cross-sectional images of exfoliated GaN/AlN membranes synthesized via 2D-assisted epitaxy on MAG-treated FLG/SiC (A) and MLG/SiC (B), respectively. Complete exfoliation was successfully achieved in both cases using a Ni stressor layer. Notably, no observable difference in exfoliation behavior or surface morphology was found between the two, indicating that the applied strain energy is sufficient to detach the membrane from the graphene layer, regardless of graphene thickness. The macroscopically smooth surfaces observed in the SEM images validate the effectiveness and consistency of the 2DLT process across different graphene configurations. The scale bar in the SEM images is 500 nm.

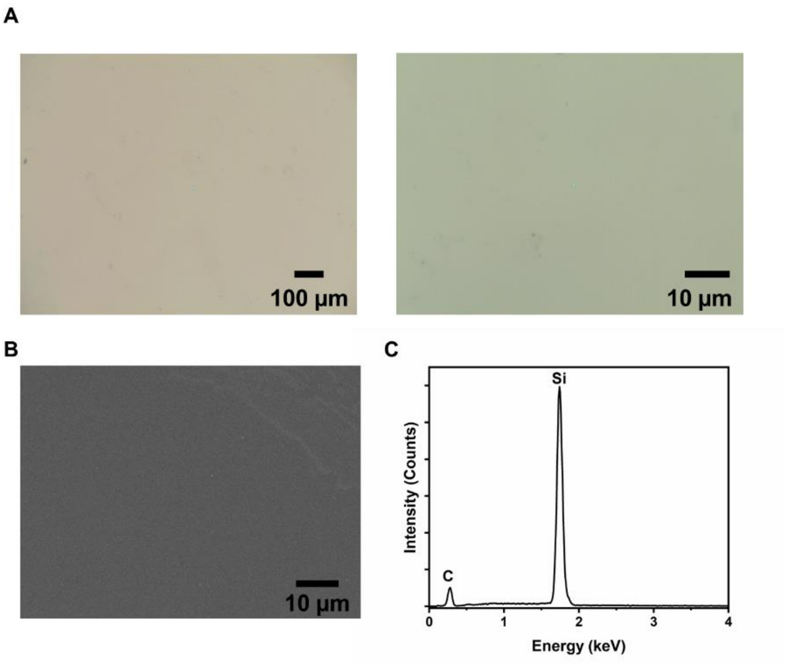

Fig. S32. Characterization of the FLG/SiC substrate surface after exfoliation of the GaN/AlN membrane. (**A**) Optical images of the SiC substrate after exfoliation show a clean surface without visible cracks or residues. (**B**) SEM image of the exfoliated substrate, where characteristic wrinkles of the underlying graphene layer are observed. The morphology closely resembles that of the pristine FLG/SiC sample, confirming that the graphene surface is preserved without damage. (**C**) EDS spectrum of the substrate surface showing only Si and C peaks, verifying that the exfoliation process cleanly separates the GaN/AlN layer and leaves the graphitized SiC substrate largely intact.

Table S1. Comparative analysis of AlN crystalline quality on MAG-treated SiC versus 2D-assisted epitaxial substrates. The table highlights the markedly improved crystalline quality of free-standing AlN grown on MAG-treated SiC, compared to other 2D-material-assisted epitaxial substrates. Notably, our graphene layer does not induce defects and can be easily transferred to other substrates, which is highly advantageous for substrate reuse and enables the use of the membrane as a freestanding device. LT refers to low-temperature growth; TDD refers to threading dislocation density; qvdWE refers to quasi-vdWE. The threading dislocation density (TDD) was calculated from XRD rocking curve data for both the (002) and (102) reflections using the Berger model.

**Material**	**2D-material**	**2D material growth method**	**Substrate**	**AlN Growth**	**(002) / (102) FWHM (arcsec)**	**TDD (cm^{-2})**	**Exfoliation**	**Ref**
AlN	Multi-layer Graphene	High temp graphitization	SiC	HVPE (vdWE)	3600 / -	-	O	(55)
	Defect induced Graphene	CVD (1055 °C, 3-5 h)	Al_2O_3	MOCVD (qvdWE)	315.5 / 518	2.7×10^9	×	(*57*)
	Defect induced Graphene	High temp graphitization (> 1600 °C)	SiC	MOCVD (qvdWE)	148 / 79	6.7×10^8	×	(*50*)
	Multi-layer Graphene	*MAG (450 °C)*	*SiC*	MOCVD (vdWE)	*1774 / 2136*	3.1×10^{10}	*O*	*This work*
	Few-layer Graphene	*MAG (320 °C)*	*SiC*	MOCVD (Remote Epitaxy)	*759 / 833*	3.8×10^9	*O*	*This work*

Table S2. Comparison of the crystalline quality of GaN/AlN heterostructures on MAG-treated SiC versus other substrates. The results suggest that our MAG-treated SiC offers a desirable platform for GaN materials. LT refers to low-temperature growth. The threading dislocation density (TDD) was calculated from XRD rocking curve data for both the (002) and (102) reflections using the Berger model.

Material	Buffer layer	Substrate	Growth Method	(002) FWHM (arcsec)	Threading Dislocation Density (cm^{-2})	Reference
GaN	AlN	Si (111)	MOCVD	380	3×10^9	(75)
	LT-AlN	Al_2O_3	MOCVD	380	6×10^8	(*60*)
	LT-GaN	Al_2O_3	MOCVD	220	9×10^8	(*60*)
	AlN / h-BN (3nm)	Al_2O_3	MOCVD	576	1.6×10^{10}	(*63*)
	AlN / h-BN	Al_2O_3	MOCVD	396	3×10^8	(*65*)
	AlN	SiC	MOCVD	200	2×10^9	(*61*)
	Graphene buffer layer	SiC	MBE	141	2.1×10^7	(*6*)
	1 ML Graphene	SiC	MBE	328	6.3×10^8	(*6*)
	LT-GaN (150 nm) / Graphene buffer layer	SiC	MOCVD	544	3.0×10^9	(*62*)
	1 ML Graphene	SiC	MOCVD	222	1×10^9	(*4*)
	AlN / Multi-layer Graphene	SiC	MOCVD	1260	1.4×10^{10}	(*64*)
	AlN / FLG	*SiC*	MOCVD	*270*	1.53×10^9	*This work*
	AlN / MLG	*SiC*	MOCVD	*444*	4.07×10^9	*This work*

Other Supplementary Materials for this manuscript include:

Movie S1. AIMD simulation of the Ni/graphitic carbon/SiC system.

Movie S2. AIMD simulation of the Fe/graphitic carbon/SiC system.

Movie S3. AIMD simulation of the Ru/graphitic carbon/SiC system.

REFERENCES AND NOTES

1. H. Kum, D. Lee, W. Kong, H. Kim, Y. Park, Y. Kim, Y. Baek, S. H. Bae, K. Lee, J. Kim, Epitaxial growth and layer-transfer techniques for heterogeneous integration of materials for electronic and photonic devices. *Nat. Electron.* **2**, 439–450 (2019).

2. H. Kim, C. S. Chang, S. Lee, J. Jiang, J. Jeong, M. Park, Y. Meng, J. Ji, Y. Kwon, X. Sun, W. Kong, H. S. Kum, S. H. Bae, K. Lee, Y. J. Hong, J. Shi, J. Kim, Remote epitaxy. *Nat. Rev. Methods Primers* **2**, 40 (2022).

3. C. S. Chang, K. S. Kim, B.-I. Park, J. Choi, H. Kim, J. Jeong, M. Barone, N. Parker, S. Lee, X. Zhang, K. Lu, J. M. Suh, J. Kim, D. Lee, N. M. Han, M. Moon, Y. S. Lee, D.-H. Kim, D. G. Schlom, Y. J. Hong, J. Kim, Remote epitaxial interaction through graphene. *Sci. Adv.* **9**, eadj5379 (2023).

4. J. Kim, C. Bayram, H. Park, C. W. Cheng, C. Dimitrakopoulos, J. A. Ott, K. B. Reuter, S. W. Bedell, D. K. Sadana, Principle of direct van der Waals epitaxy of single-crystalline films on epitaxial graphene. *Nat. Commun.* **5**, 4836 (2014).

5. J. Narayan, Recent progress in thin film epitaxy across the misfit scale. *Acta Mater.* **61**, 2703–2724 (2013).

6. H. S. Kum, H. Lee, S. Kim, S. Lindemann, W. Kong, K. Qiao, P. Chen, J. Irwin, J. H. Lee, S. Xie, S. Subramanian, J. Shim, S. H. Bae, C. Choi, L. Ranno, S. Seo, S. Lee, J. Bauer, H. Li, K. Lee, J. A. Robinson, C. A. Ross, D. G. Schlom, M. S. Rzchowski, C. B. Eom, J. Kim, Heterogeneous integration of single-crystalline complex-oxide membranes. *Nature* **578**, 75–81 (2020).

7. Y. Kim, S. S. Cruz, K. Lee, B. O. Alawode, C. Choi, Y. Song, J. M. Johnson, C. Heidelberger, W. Kong, S. Choi, K. Qiao, I. Almansouri, E. A. Fitzgerald, J. Kong, A. M. Kolpak, J. Hwang, J. Kim, Remote epitaxy through graphene enables two-dimensional material-based layer transfer. *Nature* **544**, 340–343 (2017).

8. K. Qiao, Y. Liu, C. Kim, R. J. Molnar, T. Osadchy, W. Li, X. Sun, H. Li, R. L. Myers-Ward, D. Lee, S. Subramanian, H. Kim, K. Lu, J. A. Robinson, W. Kong, J. Kim, Graphene buffer layer on SiC as a release layer for high-quality freestanding semiconductor membranes. *Nano Lett.* **21**, 4013–4020 (2021).

9. K. V. Emtsev, A. Bostwick, K. Horn, J. Jobst, G. L. Kellogg, L. Ley, J. L. McChesney, T. Ohta, S. A. Reshanov, J. Röhrl, E. Rotenberg, A. K. Schmid, D. Waldmann, H. B. Weber, T. Seyller, Towards wafer-size graphene layers by atmospheric pressure graphitization of silicon carbide. *Nat. Mater.* **8**, 203–207 (2009).

10. C. Berger, Z. Song, X. Li, X. Wu, N. Brown, C. Naud, D. Mayou, T. Li, J. Hass, A. N. Marchenkov, E. H. Conrad, P. N. First, W. A. De Heer, Electronic confinement and coherence in patterned epitaxial graphene. *Science* **312**, 1191–1196 (2006).

11. Z. Y. Juang, C. Y. Wu, C. W. Lo, W. Y. Chen, C. F. Huang, J. C. Hwang, F. R. Chen, K. C. Leou, C. H. Tsai, Synthesis of graphene on silicon carbide substrates at low temperature. *Carbon* **47**, 2026–2031 (2009).

12. C. Li, D. Li, J. Yang, X. Zeng, W. Yuan, Preparation of single- and few-layer graphene sheets using Co deposition on SiC substrate. *J. Nanomater.* **2011**, 319624 (2011).

13. F. Iacopi, N. Mishra, B. V. Cunning, D. Goding, S. Dimitrijev, R. Brock, R. H. Dauskardt, B. Wood, J. Boeckl, A catalytic alloy approach for graphene on epitaxial SiC on silicon wafers. *J. Mater. Res.* **30**, 609–616 (2015).

14. H. I. Røst, R. K. Chellappan, F. S. Strand, A. Grubisic-Cabo, B. P. Reed, M. J. Prieto, L. C. Tanase, L. De Souza Caldas, T. Wongpinij, C. Euaruksakul, T. Schmidt, A. Tadich, B. C. C. Cowie, Z. Li, S. P. Cooil, J. W. Wells, Low-temperature growth of graphene on a semiconductor. *J. Phys. Chem. C* **125**, 4243–4252 (2021).

15. E. Escobedo-Cousin, K. Vassilevski, I. Nikitina, N. Wright, A. O'Neill, A. Horsfall, J. Goss, Local solid phase epitaxy of few-layer graphene on silicon carbide. *Mater. Sci. Forum* **717-720**, 629–632 (2012).

16. P. MacHáč, T. Fidler, S. Cichoň, L. Mišková, Synthesis of graphene on SiC substrate via Ni-silicidation reactions. *Thin Solid Films* **520**, 5215–5218 (2012).

17. P. Macháč, T. Fidler, S. Cichoň, V. Jurka, Synthesis of graphene on Co/SiC structure. *J. Mater. Sci. Mater. Electron.* **24**, 3793–3799 (2013).

18. E. Escobedo-Cousin, K. Vassilevski, T. Hopf, N. Wright, A. O'Neill, A. Horsfall, J. Goss, Solid phase growth of graphene on silicon carbide by nickel silicidation: Graphene formation mechanisms. *Mater. Sci. Forum* **778-780**, 1162–1165 (2014).

19. S. Lim, J. S. Oh, Y. Kwon, B. S. An, J. H. Bae, T. H. Kim, M. H. Park, H. S. Kim, C. W. Yang, Interfacial reactions in Ni/6H-SiC at low temperatures. *J. Nanosci. Nanotechnol.* **16**, 10853–10857 (2016).

20. Y. Kwon, B. S. An, C. W. Yang, Direct observation of interfacial reaction of Ni/6H-SiC and carbon redistribution by in situ transmission electron microscopy. *Mater. Charact.* **140**, 259–264 (2018).

21. A. Hähnel, V. Ischenko, J. Woltersdorf, Oriented growth of silicide and carbon in SiC-based sandwich structures with nickel. *Mater. Chem. Phys.* **110**, 303–310 (2008).

22. J. Hofrichter, B. N. Szafranek, M. Otto, T. J. Echtermeyer, M. Baus, A. Majerus, V. Geringer, M. Ramsteiner, H. Kurz, Synthesis of graphene on silicon dioxide by a solid carbon source. *Nano Lett.* **10**, 36–42 (2010).

23. A. Delamoreanu, C. Rabot, C. Vallee, A. Zenasni, Wafer scale catalytic growth of graphene on nickel by solid carbon source. *Carbon* **66**, 48–56 (2014).

24. S. P. Cooil, F. Song, G. T. Williams, O. R. Roberts, D. P. Langstaff, B. Jørgensen, K. Høydalsvik, D. W. Breiby, E. Wahlström, D. A. Evans, J. W. Wells, Iron-mediated growth of epitaxial graphene on SiC and diamond. *Carbon* **50**, 5099–5105 (2012).

25. P. Machac, T. Hrebicek, Modification of carbon solubility in metals at preparation of graphene from the metal/SiC structure. *J. Mater. Sci. Mater. Electron.* **28**, 12425–12431 (2017).

26. T. C. Chou, A. Joshi, J. Wadsworth, Solid state reactions of SiC with Co, Ni, and Pt. *J. Mater. Res.* **6**, 796–809 (1991).

27. W. M. Tang, Z. X. Zheng, H. F. Ding, Z. H. Jin, A study of the solid state reaction between silicon carbide and iron. *Mater. Chem. Phys.* **74**, 258–264 (2002).

28. M. Wu, H. Huang, Y. Wu, X. Wu, Mechanism of solid-state diffusion reaction in vacuum between metal (Fe, Ni, and Co) and 4H–SiC. *Ceram. Int.* **50**, 17930–17939 (2024).

29. M. E. Schlesinger, Thermodynamics of solid transition-metal silicides. *Chem. Rev.* **90**, 607–628 (1990).

30. B. P. Nash, A. Nash, The Ni–Si (nickel–silicon) system equilibrium diagram. *Bull. Alloy Phase Diagrams* **8**, 6–14 (1987).

31. L. Perring, F. Bussy, J. C. Gachon, P. Feschotte, The ruthenium–silicon system. *J. Alloys Compd.* **284**, 198–205 (1999).

32. C. Mattevi, H. Kim, M. Chhowalla, A review of chemical vapour deposition of graphene on copper. *J. Mater. Chem.* **21**, 3324–3334 (2011).

33. Y. Cao, L. Nyborg, U. Jelvestam, XPS calibration study of thin-film nickel silicides. *Surf. Interface Anal.* **41**, 471–483 (2009).

34. N. Ohtsu, M. Oku, K. Satoh, K. Wagatsuma, Dependence of core-level XPS spectra on iron silicide phase. *Appl. Surf. Sci.* **264**, 219–224 (2013).

35. S. van Vliet, A. Troglia, E. Olsson, R. Bliem, Identifying silicides via plasmon loss satellites in photoemission of the Ru-Si system. *Appl. Surf. Sci.* **608**, 155139 (2023).

36. Y. Hoshino, S. Matsumoto, T. Nakada, Y. Kido, Interfacial reactions between ultra-thin Ni-layer and clean 6H-SiC(0001) surface. *Surf. Sci.* **556**, 78–86 (2004).

37. K. V. Emtsev, F. Speck, T. Seyller, L. Ley, J. D. Riley, Interaction, growth, and ordering of epitaxial graphene on SiC{0001} surfaces: A comparative photoelectron spectroscopy study. *Phys. Rev. B* **77**, 155303 (2008).

38. N. Zebardastan, J. Bradford, J. Lipton-Duffin, J. MacLeod, K. Ostrikov, M. Tomellini, N. Motta, High quality epitaxial graphene on 4H-SiC by face-to-face growth in ultra-high vacuum. *Nanotechnology* **34**, 105601 (2023).

39. J. Röhrl, M. Hundhausen, K. V. Emtsev, T. Seyller, R. Graupner, L. Ley, Raman spectra of epitaxial graphene on SiC(0001). *Appl. Phys. Lett.* **92**, 201918 (2008).

40. Z. H. Ni, W. Chen, X. F. Fan, J. L. Kuo, T. Yu, A. T. S. Wee, Z. X. Shen, Raman spectroscopy of epitaxial graphene on a SiC substrate. *Phys. Rev. B* **77**, 115416 (2008).

41. A. Eckmann, A. Felten, A. Mishchenko, L. Britnell, R. Krupke, K. S. Novoselov, C. Casiraghi, Probing the nature of defects in graphene by Raman spectroscopy. *Nano Lett.* **12**, 3925–3930 (2012).

42. A. Zandiatashbar, G. H. Lee, S. J. An, S. Lee, N. Mathew, M. Terrones, T. Hayashi, C. R. Picu, J. Hone, N. Koratkar, Effect of defects on the intrinsic strength and stiffness of graphene. *Nat. Commun.* **5**, 3186 (2014).

43. T. M. G. Mohiuddin, A. Lombardo, R. R. Nair, A. Bonetti, G. Savini, R. Jalil, N. Bonini, D. M. Basko, C. Galiotis, N. Marzari, K. S. Novoselov, A. K. Geim, A. C. Ferrari, Uniaxial strain in graphene by Raman spectroscopy: G peak splitting, Grüneisen parameters, and sample orientation. *Phys. Rev. B* **79**, 205433 (2009).

44. E. B. Barros, N. S. Demir, A. G. Souza Filho, J. Mendes Filho, A. Jorio, G. Dresselhaus, M. S. Dresselhaus, Raman spectroscopy of graphitic foams. *Phys. Rev. B* **71**, 165422 (2005).

45. J. A. Robinson, C. P. Puls, N. E. Staley, J. P. Stitt, M. A. Fanton, K. V. Emtsev, T. Seyller, Y. Liu, Raman topography and strain uniformity of large-area epitaxial graphene. *Nano Lett.* **9**, 964–968 (2009).

46. Y. Hao, Y. Wang, L. Wang, Z. Ni, Z. Wang, R. Wang, C. K. Koo, Z. Shen, J. T. L. Thong, Probing layer number and stacking order of few-layer graphene by Raman spectroscopy. *Small* **6**, 195–200 (2010).

47. A. C. Ferrari, D. M. Basko, Raman spectroscopy as a versatile tool for studying the properties of graphene. *Nat. Nanotechnol.* **8**, 235–246 (2013).

48. W. Kong, H. Li, K. Qiao, Y. Kim, K. Lee, Y. Nie, D. Lee, T. Osadchy, R. J. Molnar, D. K. Gaskill, R. L. Myers-Ward, K. M. Daniels, Y. Zhang, S. Sundram, Y. Yu, S. H. Bae, S. Rajan, Y. Shao-Horn, K. Cho, A. Ougazzaden, J. C. Grossman, J. Kim, Polarity governs atomic interaction through two-dimensional materials. *Nat. Mater.* **17**, 999–1004 (2018).

49. S. H. Bae, K. Lu, Y. Han, S. Kim, K. Qiao, C. Choi, Y. Nie, H. Kim, H. S. Kum, P. Chen, W. Kong, B. S. Kang, C. Kim, J. Lee, Y. Baek, J. Shim, J. Park, M. Joo, D. A. Muller, K. Lee, J. Kim, Graphene-assisted spontaneous relaxation towards dislocation-free heteroepitaxy. *Nat. Nanotechnol.* **15**, 272–276 (2020).

50. Y. Wang, S. Yang, H. Chang, W. Li, X. Chen, R. Hou, J. Yan, X. Yi, J. Wang, T. Wei, Flexible graphene-assisted van der Waals epitaxy growth of crack-free AlN epilayer on SiC by lattice engineering. *Appl. Surf. Sci.* **520**, 146358 (2020).

51. D. K. Pradhan, D. C. Moore, A. M. Francis, J. Kupernik, W. J. Kennedy, N. R. Glavin, R. H. Olsson, D. Jariwala, Materials for high-temperature digital electronics. *Nat. Rev. Mater.* **9**, 790–807 (2024).

52. C. Zhou, A. Ghods, V. G. Saravade, P. V. Patel, K. L. Yunghans, C. Ferguson, Y. Feng, B. Kucukgok, N. Lu, I. T. Ferguson, Review—The current and emerging applications of the III-nitrides. *ECS J. Solid State Sci. Technol.* **6**, Q149–Q156 (2017).

53. J. Gong, J. Zhou, P. Wang, T. H. Kim, K. Lu, S. Min, R. Singh, M. Sheikhi, H. N. Abbasi, D. Vincent, D. Wang, N. Campbell, T. Grotjohn, M. Rzchowski, J. Kim, E. T. Yu, Z. Mi, Z. Ma, Synthesis and characteristics of transferrable single-crystalline AlN nanomembranes. *Adv. Electron. Mater.* **9**, 2201309 (2023).

54. J. Y. Tsao, S. Chowdhury, M. A. Hollis, D. Jena, N. M. Johnson, K. A. Jones, R. J. Kaplar, S. Rajan, C. G. Van de Walle, E. Bellotti, C. L. Chua, R. Collazo, M. E. Coltrin, J. A. Cooper, K. R. Evans, S. Graham, T. A. Grotjohn, E. R. Heller, M. Higashiwaki, M. S. Islam, P. W. Juodawlkis, M. A. Khan, A. D. Koehler, J. H. Leach, U. K. Mishra, R. J. Nemanich, R. C. N. Pilawa-Podgurski, J. B. Shealy, Z. Sitar, M. J. Tadjer, A. F. Witulski, M. Wraback, J. A. Simmons, Ultrawide-bandgap semiconductors: Research opportunities and challenges. *Adv. Electron. Mater.* **4**, 1600501 (2018).

55. Y. Xu, B. Cao, Z. Li, D. Cai, Y. Zhang, G. Ren, J. Wang, L. Shi, C. Wang, K. Xu, Growth model of van der Waals epitaxy of films: A case of AlN films on multilayer graphene/SiC. *ACS Appl. Mater. Interfaces* **9**, 44001–44009 (2017).

56. F. Liu, T. Wang, X. Gao, H. Yang, Z. Zhang, Y. Guo, Y. Yuan, Z. Huang, J. Tang, B. Sheng, Z. Chen, K. Liu, B. Shen, X.-Z. Li, H. Peng, X. Wang, Determination of the preferred epitaxy for III-nitride semiconductors on wet-transferred graphene. *Sci. Adv.* **9**, eadf8484 (2023).

57. Z. Chen, Z. Liu, T. Wei, S. Yang, Z. Dou, Y. Wang, H. Ci, H. Chang, Y. Qi, J. Yan, J. Wang, Y. Zhang, P. Gao, J. Li, Z. Liu, Improved epitaxy of AlN film for deep-ultraviolet light-emitting diodes enabled by graphene. *Adv. Mater.* **31**, e1807345 (2019).

58. P. Avouris, C. Dimitrakopoulos, Graphene: Synthesis and applications. *Mater. Today* **15**, 86–97 (2012).

59. W. Chen, M. A. Capano, Growth and characterization of 4H-SiC epilayers on substrates with different off-cut angles. *J. Appl. Phys.* **98**, 114907 (2005).

60. C. Bayram, J. L. Pau, R. McClintock, M. Razeghi, Delta-doping optimization for high-quality p-type GaN. *J. Appl. Phys.* **104**, 083512 (2008).

61. Z. J. Reitmeier, S. Einfeldt, R. F. Davis, X. Zhang, X. Fang, S. Mahajan, Surface and defect microstructure of GaN and AlN layers grown on hydrogen-etched 6H-SiC(0001) substrates. *Acta Mater.* **58**, 2165–2175 (2010).

62. S. Lee, J. Kim, B. I. Park, H. I. Kim, C. Lim, E. Lee, J. Y. Yang, J. Choi, Y. J. Hong, C. S. Chang, H. S. Kum, J. Kim, K. Lee, H. Kim, G. C. Yi, GaN remote epitaxy on a pristine graphene buffer layer via controlled graphitization of SiC. *Appl. Phys. Lett.* **125**, 252102 (2024).

63. Y. Kobayashi, K. Kumakura, T. Akasaka, T. Makimoto, Layered boron nitride as a release layer for mechanical transfer of GaN-based devices. *Nature* **484**, 223–227 (2012).

64. Y. Yu, T. Wang, X. Chen, L. Zhang, Y. Wang, Y. Niu, J. Yu, H. Ma, X. Li, F. Liu, G. Deng, Z. Shi, B. Zhang, X. Wang, Y. Zhang, Demonstration of epitaxial growth of strain-relaxed GaN films on graphene/SiC substrates for long wavelength light-emitting diodes. *Light Sci. Appl.* **10**, 117 (2021).

65. N. R. Glavin, K. D. Chabak, E. R. Heller, E. A. Moore, T. A. Prusnick, B. Maruyama, D. E. Walker, D. L. Dorsey, Q. Paduano, M. Snure, Flexible gallium nitride for high-performance, strainable radio-frequency devices. *Adv. Mater.* **29**, 1701838 (2017).

66. G. Kresse, J. Furthmüller, Efficient iterative schemes for ab initio total-energy calculations using a plane-wave basis set. *Phys. Rev. B* **54**, 11169–11186 (1996).

67. P. E. Blöchl, Projector augmented-wave method. *Phys. Rev. B* **50**, 17953–17979 (1994).

68. J. P. Perdew, K. Burke, M. Ernzerhof, Generalized gradient approximation made simple. *Phys. Rev. Lett.* **77**, 3865–3868 (1996).

69. G. Makov, M. C. Payne, Periodic boundary conditions in ab initio calculations. *Phys. Rev. B* **51**, 4014–4022 (1995).

70. S. Nosé, A unified formulation of the constant temperature molecular dynamics methods. *J. Chem. Phys.* **81**, 511–519 (1984).

71. W. G. Hoover, Canonical dynamics: Equilibrium phase-space distributions. *Phys. Rev. A* **31**, 1695–1697 (1985).

72. D. Dardzinski, M. Yu, S. Moayedpour, N. Marom, Best practices for first-principles simulations of epitaxial inorganic interfaces. *J. Phys. Condens. Matter* **34**, 233002 (2022).

73. K. Vojtěchovský, T. Zemčík, Mössbauer study of the Fe–Si intermetallic compounds. *Czech. J. Phys. B* **24**, 171–178 (1974).

74. R. R. Nair, P. Blake, A. N. Grigorenko, K. S. Novoselov, T. J. Booth, T. Stauber, N. M. R. Peres, A. K. Geim, Fine structure constant defines visual transparency of graphene. *Science* **320**, 1308 (2008).

75. E. Arslan, M. K. Ozturk, A. Teke, S. Ozcelik, E. Ozbay, Buffer optimization for crack-free GaN epitaxial layers grown on Si(111) substrate by MOCVD. *J. Phys. D Appl. Phys.* **41**, 155307 (2008).
